# Three-dimensional nanofibrous PCL/gelatin scaffold fabricated using centrifugal force assisted wet electrospinning technique

**DOI:** 10.1038/s41598-025-20814-z

**Published:** 2025-10-23

**Authors:** Sogand Abedi, Atieh Mohajeri, Soheila Zamanlui Benisi, Mohamad Pezeshki-Modaress, Salar Mohammadi Shabestari

**Affiliations:** 1 Department of Biology, Faculty of Basic Sciences, Central Tehran Branch, Islamic Azad University, P.O. Box 13145-784, Tehran, Iran; 2 Department of Biomedical Engineering, CT.C., Islamic Azad University, P.O. Box 13185/768, Tehran, Iran; 3 Burn Research Center, Iran University of Medical Sciences, Tehran, Iran; 4 Department of Polymer, School of Chemical Engineering, College of Engineering, University of Tehran, Tehran, Iran; 5 Department of Plastic and Reconstructive surgery, Hazrat Fatemeh Hospital, School of Medicine, Iran University of Medical Sciences, Tehran, Iran

**Keywords:** Wet electrospinning, Centrifugation, Porosity, 3D scaffold, C6 glial cell line, Neural regeneration, Biochemistry, Biological techniques, Biotechnology

## Abstract

A three-dimensional (3D) scaffold that enables optimal cell–matrix interactions is essential for developing physiologically relevant neural tissue models. In this study, wet electrospinning was optimized to fabricate nanofibrous PCL/gelatin scaffolds with a well-controlled 3D architecture, using centrifugal force to tune scaffold morphology, porosity, and mechanical properties. The effects of centrifugal force intensity (5000 vs. 10,000 rpm) and application time (5 vs. 10 min) were systematically investigated. Scaffolds fabricated at 5000 rpm exhibited poor structural integrity and were excluded from further analysis. Among scaffolds produced at 10,000 rpm, blends of PCL/gelatin at 70:30 and 60:40 demonstrated excellent porosity (98.1 ± 1.9% and 97.3 ± 1.1%, respectively) and favorable fiber architecture. The 70:30–10 min scaffold achieved the highest tensile strength (57.03 ± 1.50 kPa) and modulus (53.00 ± 2.00 kPa), aligning with the physiological range of neural tissues. MTT assays confirmed robust biocompatibility, with C6 glial cell viability increasing by + 4.30% on the 70:30–10 min scaffold and + 5.88% on the 60:40–10 min scaffold over 14 days. Although the 70:30–5 min scaffold showed the highest proliferation (+ 12.16%), the 10-min variant was selected for detailed morphological evaluation due to its superior mechanical performance and structural uniformity. DAPI and H&E staining further validated the enhanced cell aggregation, ECM deposition, and neural-like morphology within the 70:30–10 min scaffold. These results collectively highlight the critical role of scaffold composition and processing parameters in engineering 3D neural tissue scaffolds, with the optimized 70:30–10 min scaffold emerging as a promising candidate for advanced neural tissue engineering applications.

## Introduction

The scaffold architecture is a critical determinant of cell fate in therapeutic strategies, particularly through its influence on porosity, fiber orientation, mechanical stiffness, and biodegradation kinetics. These structural parameters play a central role in regulating cell adhesion, migration, proliferation, and lineage specification, which are essential for effective tissue regeneration^1^. Nanotechnology has expanded the potential of scaffold design by enabling control over topographical, mechanical, and biochemical cues at the nanoscale, leading to more accurate emulation of the native extracellular matrix (ECM) and improvement in cell–matrix interactions^2^. Among various tissue engineering applications, neural tissue regeneration remains one of the most challenging due to the intricate architecture and highly specialized cellular microenvironment of the central nervous system. Neural cells are extremely sensitive to the biochemical and biophysical properties of their surrounding matrix, requiring scaffolds that not only support cell survival and proliferation but also promote alignment, neurite outgrowth, and ECM deposition^3^. Recent advances in biomaterials have enabled the design of scaffolds that closely mimic the physicochemical properties of the native ECM, not only in morphology but also in functional bioactivity^4^. For example, silk-based conductive composites have demonstrated superior biointerface performance by integrating electrical responsiveness with ECM-like flexibility and biocompatibility, offering new opportunities for neural and cardiac regeneration applications^5^. Similarly, piezoelectric nanofibers fabricated via electrospinning have shown promise in mediating self-powered electrical stimulation to enhance tissue regeneration, particularly in electro-sensitive tissues such as nerve and muscle^6^. These materials exemplify the growing trend toward multifunctional scaffolds that provide mechanical support, topographical cues, and bioelectrical modulation simultaneously.

Electrospinning is one of the most versatile and widely employed techniques for fabricating nanofibrous scaffolds. It enables the production of fibers with diameters ranging from a few nanometers to several micrometers, with morphological features that mimic the fibrillar components of natural ECM^7,8^. Electrospun scaffolds exhibit high surface-to-volume ratios, tunable pore sizes, and high porosity, which are beneficial for nutrient diffusion, waste elimination, and cellular ingrowth. Nevertheless, conventional dry electrospinning is limited by its tendency to form dense, two-dimensional (2D) fiber mats with low thickness and limited pore interconnectivity, restricting deep cellular penetration and three-dimensional (3D) tissue formatio^9–11^. This structural limitation poses a critical barrier in applications such as neural tissue engineering, where scaffold architecture must support the migration, aggregation, and neurite extension of glial and neuronal cells in all directions^12^.

To overcome these shortcomings, wet electrospinning has been introduced as a modified process in which fibers are deposited into a coagulation bath composed of a non-solvent medium. This approach prevents the compact accumulation of fibers observed in dry collection, allowing the formation of loosely packed, volumetric, and interconnected fibrous networks^13^. The bath medium also serves as a sink for solvent removal, reducing cytotoxicity and enhancing biocompatibility^14^. Moreover, wet electrospinning facilitates greater control over scaffold geometry, fiber orientation, and porosity, making it particularly attractive for applications requiring biomimetic microenvironments^15^. The resulting 3D structures more accurately reflect the topography and mechanics of in vivo tissue matrices and allow better cell infiltration and nutrient transport^14^.

Among other fabrication technologies, 3D bioprinting has gained attention for its ability to create scaffolds with precise macroscopic architectures through computer-aided design^16^. However, bioprinting generally lacks the resolution necessary to mimic ECM nanostructures and often produces constructs with inadequate mechanical strength and low structural fidelity at the microscale. The quality of bioprinted scaffolds is highly dependent on the rheological properties and gelation behavior of the bioinks, which can vary significantly between formulations and conditions^17^. Furthermore, the time-consuming printing process, cell viability challenges, and high cost of equipment and materials limit the scalability of this method for widespread biomedical applications ^18^.

In contrast, centrifugal force-assisted wet electrospinning, as used in the present study, introduces a novel paradigm by combining the nanoscale control of electrospinning with enhanced spatial architecture through external mechanical modulation. Centrifugal force contributes to uniform fiber deposition and increased scaffold thickness by directing the orientation and distribution of fibers within the coagulation bath^19^. By adjusting parameters such as spinning time and speed (revolutions per minute), it is possible to tune the scaffold’s porosity, density, and mechanical strength with high precision. This synergistic method enables fabrication of scaffolds with superior thickness, open porosity (> 90%), and improved vertical alignment compared to standard electrospinning approaches^20^.

A key novelty of this method lies in the dynamic balance between electrostatic fiber elongation and spatial rearrangement modulated by gravity and centrifugal acceleration. This dual control provides an additional axis for tailoring fiber layering, pore connectivity, and scaffold thickness—features that conventional dry electrospinning or even advanced 3D bioprinting do not fully achieve. Such control is particularly relevant in neural tissue engineering, where scaffolds must simultaneously combine high porosity for nutrient transport with neural-compatible mechanics in the kilopascal range, and promote topographical cues guiding axonal pathfinding and glial alignment^21^. The integration of gravitational and centrifugal shaping in wet electrospinning has been rarely explored in the literature and represents a largely untapped approach with significant translational potential. To place this strategy in context, Table 1 provides a comparative overview of scaffold fabrication methods, highlighting how centrifugal wet electrospinning bridges the gap between nanoscale resolution and three-dimensional structural fidelity. A more detailed benchmarking against prior studies, including quantitative mechanical, porosity, and biological endpoints, is provided in the Discussion section to articulate the novelty of the present work^19,22–24^.

**Table 1:** Comparative overview of scaffold fabrication techniques highlighting the advantages of centrifugal wet electrospinning in combining nanoscale resolution with 3D structural control and tunable mechanical performance.

Feature/parameter	Dry electrospinning	3D bioprinting	Centrifugal wet electrospinning (this study)	Refs.
Fiber scale/resolution	Nano to microscale	Microscale (limited nanoscale)	Nanoscale fibers with uniform deposition	^24^
Porosity and interconnectivity	Low; dense 2D mats	High; customizable macro-porosity	High (> 90%); open and interconnected	^19^
Scaffold thickness	Low (~ < 500 µm)	Tunable (mm to cm)	Moderate-to-high (up to mm-scale)	^23^
Mechanical strength	Moderate	Often low	Tunable; enhanced with centrifugation	^24^
ECM-like topography	Yes (at fiber scale)	Limited	Yes; with 3D spatial fiber orientation	^22^
Cell infiltration capacity	Limited (surface-level only)	High	High (due to 3D porosity and geometry)	^23^
Speed and scalability	High throughput	Time-consuming	High (centrifugal acceleration reduces time)	^19^
Material compatibility	Wide range	Limited by bioink properties	Wide (PCL, gelatin, blends, etc.)	^24^
Cost and complexity	Low	High (equipment + material)	Moderate; uses basic centrifuge & spinneret	^22^
Novel control mechanisms	Electrostatic only	CAD-based patterning	Electrostatic + gravitational + centrifugal shaping	^23^

Material selection plays a critical role in scaffold performance. Poly(ε-caprolactone) (PCL), a semicrystalline, biodegradable aliphatic polyester, is widely recognized for its slow degradation rate, mechanical robustness, and ease of processing^25^. However, its hydrophobic nature limits initial cell attachment and protein adsorption^26^. To address this, gelatin, a denatured form of collagen rich in cell-recognition sequences (e.g., RGD motifs), is incorporated into the blend to improve hydrophilicity, biocompatibility, and cell adhesion^27,28^. The synergistic combination of PCL and gelatin results in hybrid scaffolds with balanced mechanical properties, surface wettability, and degradation profiles suitable for neural tissue environments^29^.

Additionally, the fibrous nature of these scaffolds promotes ECM-like interactions via nanoscale topography that can activate mechanotransduction pathways such as integrin-FAK signaling^30,31^. This is particularly relevant for glial cell morphology and behavior, as the arrangement and diameter of fibers have been shown to regulate spindle-shaped cell alignment, aggregation, and neurite outgrowth^14^. Moreover, highly porous structures facilitate the exchange of oxygen, glucose, and growth factors while preventing the accumulation of metabolic waste—a key factor in maintaining cell viability in long-term cultures^32^.

Previous studies have demonstrated the potential of wet electrospun PCL-based scaffolds for tissue engineering applications. Jiang et al.^21^ reported improved alignment and elongation of vascular cells using PCL/gelatin/CNT scaffolds fabricated via wet electrospinning^15^. Similarly, Naseri-Nosar et al.^33^ showed enhanced neural regeneration in animal models using cellulose acetate/gelatin scaffolds with controlled drug release. However, the current literature has not adequately addressed the impact of external mechanical forces, such as centrifugation, on scaffold uniformity, porosity modulation, and cell response. While previous studies have largely focused on fiber morphology or drug delivery properties, the critical interplay between centrifugal shaping, scaffold architecture, and neural cell performance remains underexplored.

In this study, hybrid PCL/gelatin scaffolds using centrifugal force-assisted wet electrospinning were developed and optimized. The fabrication parameters, including polymer ratio (50:50, 60:40, and 70:30) and centrifugation conditions (speed and time), were systematically investigated to determine their effects on scaffold morphology, porosity, mechanical properties, and cellular behavior. The results confirmed that this method enables the formation of nanofibrous 3D scaffolds with interconnected pores, suitable mechanical integrity, and excellent biocompatibility for C6 glial cells. Furthermore, the biological performance of these scaffolds was comprehensively evaluated through a combination of viability assays, morphological analyses, and ECM deposition studies, providing new insights into their suitability for neural tissue engineering. This approach provides a scalable and tunable platform for engineering next-generation scaffolds for neural repair and other soft tissue applications.

## Methodology

### Materials

PCL with a molecular weight of 80,000 Da and bovine-derived gelatin were obtained from Sigma-Aldrich. Acetic acid, formic acid, ethanol, hydrochloric acid, and glutaraldehyde (Merck) were used as solvents and cross-linking agents. Fetal bovine serum (FBS), high-glucose cell culture media, and trypsin were procured from Gibco, while 4′,6-diamidino-2-phenylindole (DAPI), 3-[4,5-dimethylthiazol-2-yl]-2,5-diphenyl tetrazolium bromide (MTT), and hematoxylin and eosin (H&E) stains were supplied by Sigma. C6 rat glial cells (derived from rat glioma, ATCC® CCL-107™ equivalent) were purchased from the Pasteur Institute of Iran (Tehran, Iran). Cells were expanded and maintained in standard culture conditions following the supplier’s recommendations.

### Scaffold fabrication

**Fig. 1:**
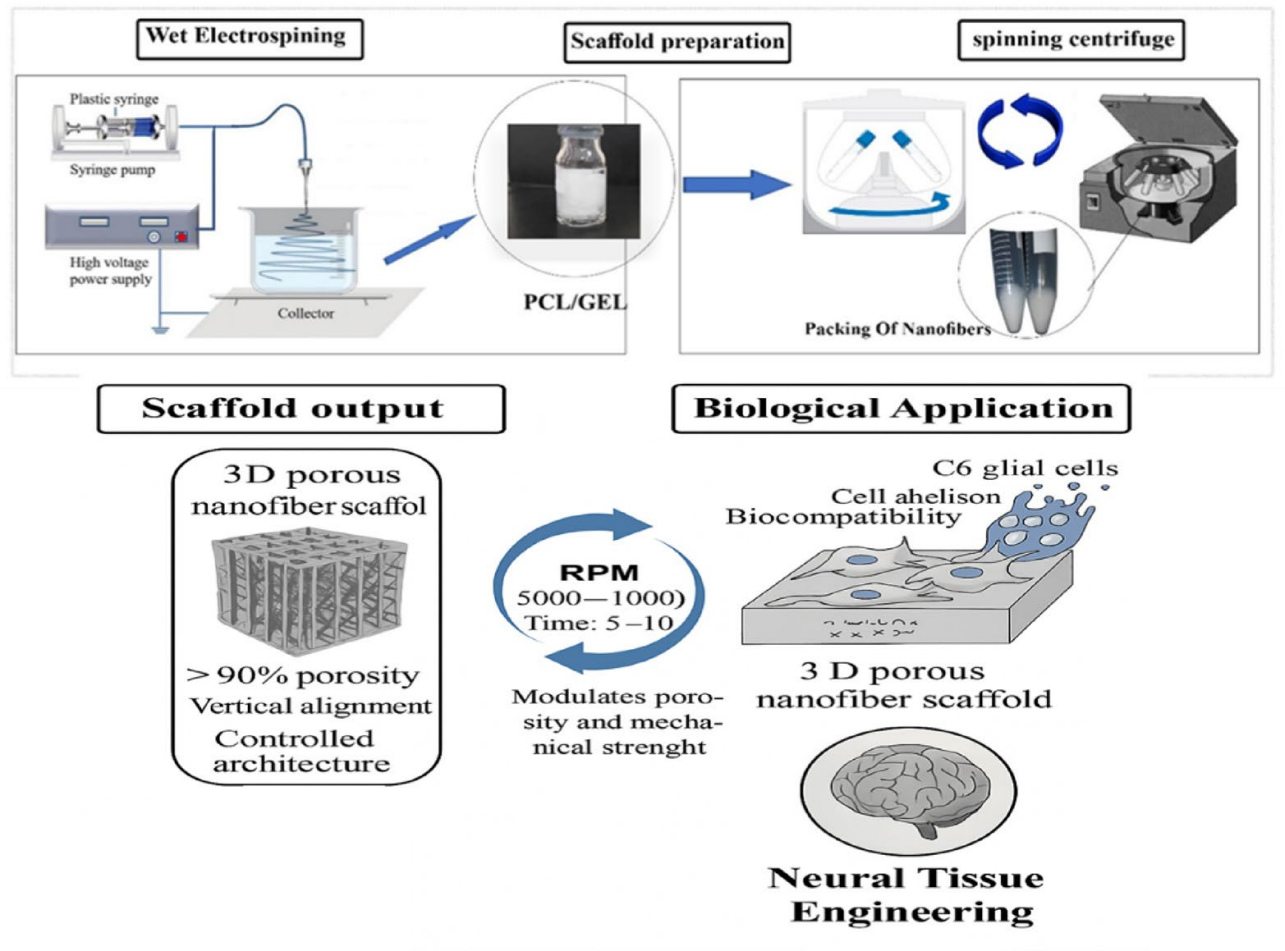
A view of employed wet electrospinning apparatus in this study and a macroscopy observation of 3D PCL/gelatin scaffold.

Scaffolds, including both pure and hybrid types, were fabricated using a wet electrospinning apparatus, as shown in Fig. 1. To prepare wet electrospun scaffolds from the PCL, the polymer was dissolved at a 15% concentration in a solvent mixture of acetic acid and formic acid at a ratio of 90:10. Hybrid scaffolds incorporating gelatin were prepared using PCL-to-gelatin ratios of 50:50, 60:40, and 70:30, respectively. The detailed parameters for wet electrospinning are presented in Table 2. The wet electrospinning process was performed under an applied voltage of 26.5 kV, with a syringe pump flow rate of 0.8 mL/h. The tip-to-collector distance, measured from the nozzle tip to the ethanol bath surface, was set to 11 cm to allow partial solvent evaporation before fiber deposition. The ethanol bath contained 250 mL of absolute ethanol in a crystallizer (150 mm diameter) at 25 ± 1 °C. Deposition time ranged from 20 to 30 min, depending on the desired scaffold thickness. Centrifugation parameters were systematically varied across polymer blend ratios (50:50, 60:40, 70:30 PCL/gelatin) at two rotation speeds (5000 and 10,000 rpm) and durations (5 and 10 min), as summarized in Table 2. This systematic variation allowed assessment of the influence of polymer composition and compaction intensity on scaffold structural and mechanical properties.

**Table 2:** The polymer concentrations for the wet electrospinning process and also, centrifugation time and speed are summarized.

PCL/gelatin ratio	Centrifuge rate (rpm)	Centrifuge time (min)
50:50	5000	5
50:50	5000	10
50:50	10,000	10
50:50	10,000	5
60:40	5000	5
60:40	5000	10
60:40	10,000	5
60:40	10,000	10
70:30	5000	5
70:30	5000	10
70:30	10,000	5
70:30	10,000	10

In this study, the collected fiber assemblies were sequentially washed with ethanol–water solutions (50:50, 30:70, and finally 0:100) to ensure complete removal of residual solvents. The washed scaffolds were subsequently subjected to centrifugation under the defined conditions, enabling controlled fiber compaction while preserving the porous architecture. Finally, the compacted scaffolds were freeze-dried at − 24 °C to eliminate residual moisture and obtain stable structures.

No chemical crosslinking was applied during or after scaffold fabrication. The structural stability of gelatin within the nanofibrous network was achieved through physical entanglement with the semi-crystalline PCL phase during centrifugal wet electrospinning, eliminating the need for potentially cytotoxic crosslinking agents. The collector bath consisted of absolute ethanol contained in a crystallizer (250 mL volume, 150 mm diameter) maintained at ambient laboratory temperature (25 ± 1 °C) with a neutral pH of 6.9 ± 0.1. Absolute ethanol was selected to minimize gelatin swelling or dissolution during fiber deposition, facilitate rapid solvent exchange, and preserve the nanofibrous morphology of the PCL/gelatin scaffolds. These conditions were kept constant throughout fabrication to ensure reproducibility. All electrospinning experiments were conducted under controlled ambient laboratory conditions (25 ± 1 °C, relative humidity 45 ± 5%) to ensure consistency in fiber morphology and scaffold properties across all fabrication batches.

The initial height of the nanofibrous mats in the Falcon tube immediately after collection in the bath was approximately 60 ± 2 mm. Following the centrifugal compaction step (10,000 rpm) and freeze-drying, the scaffold height was reduced to 10 ± 1 mm for samples with a 14 mm diameter, corresponding to an ~ 83% decrease in height. This reduction resulted from gravitational and centrifugal forces promoting fiber densification and solvent removal. These fabrication parameters were optimized to ensure uniform nanofiber deposition, minimize defects, and tailor scaffold architecture for subsequent mechanical and biological evaluations.

### SEM morphology examinations

The structure of the resulting fibers was examined with a scanning electron microscope (SEM, Philips XL30, Netherlands). The scaffold groups contained different ratios of PCL and gelatin at 50:50, 60:40 and 70:30. After freeze drying, the samples were mounted on the slides and then, coated with gold by using a sputter (Desk Sputter Coater DSR1, Nanostructural Coating Co., Iran) at a voltage of 25 kV. The specimens were then examined by SEM and the average diameter of the fibers was extracted.

### Porosity measurements

The porosity values of the prepared PCL/gelatin scaffolds were determined using pure ethanol absorption based on Archimedes’ principle. Lyophilized samples were initially weighed and then incubated in absolute ethanol for 24 h. After incubation, the wet samples were weighed, and the porosity percentages were calculated using the following formula^34^:

$${\text{Porosity}}\left( \% \right) = \frac{{W_{w} - W_{d} }}{{W_{w} - W_{l} }}\, \times \,{1}00.$$

The symbols in the porosity formula represent W_w_ as the wet weight of the scaffold after liquid absorption, W_d_ as the dry weight of the scaffold before immersion, and W_l_ as the weight of the displaced liquid, collectively used to determine the scaffold’s porosity based on its liquid absorption capacity.

### PBS uptake

The dry weight of the samples was measured after 24 h in a vacuum oven. Each sample was then immersed in phosphate buffer solution (PBS), and the wet weight was recorded at different time points until it stabilized. PBS uptake was evaluated by immersing the scaffolds in PBS at four-time intervals (15 min, 30 min, 1 h, and 4 h) at 37 °C. After immersion, excess surface liquid was removed using filter paper, and the samples were weighed. All experiments were conducted in triplicate. The PBS content in the swollen scaffolds was calculated using the following equation, where *W*_d_ and *W*_w_ represent the weights of the dry and wet scaffolds, respectively^25^:

$$PBS Uptake\left( \% \right) = \frac{{W_{w} - W_{d} }}{{W_{d} }} \times 100$$

### Mechanical evaluation

The mechanical resistance of the scaffolds to loading forces was evaluated using a universal testing machine (SANTAM, STM-20, Iran). Circular specimens with a diameter of 14 mm and a height of 1 cm were prepared for testing. The samples were compressed at a rate of 5 mm/min until a 70% reduction in volume was achieved. Each group was tested in triplicate to ensure accuracy. The resulting stress–strain curves were plotted, and the slope of the linear region was used to determine Young’s modulus (E).

### Cell culture & cell proliferation assay

For cell culture on the scaffolds, the samples were sterilized using 70% filtered ethanol for 40 min, followed by UV radiation for an additional 20 min. The scaffolds were then incubated with cell culture medium for 24 h to ensure sterility. The cell source was the C6 glial cell line, obtained from the Pasteur Institute of Iran, Tehran. After cultivation through three passages, the cells were detached using trypsin, counted, and approximately 1000 cells were seeded onto each scaffold with a diameter of 14 mm. After 25 min of cell culture, the medium was carefully removed.

The morphology of the cells cultured on the scaffolds was evaluated using SEM at 1, 7, and 14 days. For this assay, the cell-seeded scaffolds were fixed with 4% glutaraldehyde for 40 min and washed several times. The samples were then dehydrated using a graded ethanol series (50%, 60%, 70%, 80%, 90%, and 100%) with each step lasting 20 min. Finally, the dry samples were coated with gold and examined using SEM.

To evaluate scaffold toxicity, an MTT assay was performed at 1, 7, and 14 days after cell seeding. At each time point, the cell culture medium was removed, and an MTT solution (0.5 mg/mL) in medium without FBS was added. After 3 h of incubation, the MTT solution was aspirated, and DMSO was added to dissolve the formazan crystals. The absorbance values were measured at 570 nm to determine cell viability using the following formula^35,36^:

$${\text{Cell viability }}\% = \frac{OD of test group}{{OD of TCPS}}\, \times \,{1}00.$$

Here, OD is abbreviated of optical density at 570 nm and the test group defines the scaffold groups. Also, TCPS is the abbreviation for tissue culture polystyrene.

### Cell staining methods

Cell nuclear staining with 4′,6-diamidino-2-phenylindole (DAPI, Sigma) was performed to confirm cell adhesion to the scaffolds. For this assay, after 7 and 14 days of cell seeding, the cell culture medium was discarded, and the cells were fixed with 10% formalin for 2 h. Subsequently, the samples were dehydrated through a graded ethanol series ranging from 50 to 100%, with each step lasting 20 min. Paraffin was then used to embed the samples, which were sectioned into 5 µm slices using a microtome device (Leitz 1600 Saw Operative Dental Microtome, Ernst Leitz, Wetzlar, Germany). DAPI solution (0.1 µg/ml) was applied to the sections for 150 s, and the stained cells were observed under a fluorescence microscope (Nikon, Eclipse TE2000-S, Japan).

In addition, hematoxylin–eosin (H&E) staining was conducted to further assess cell morphology. The cell nuclei were stained with hematoxylin solution for 4 min, followed by washing with 0.3% hydrochloric acid/ethanol and water. Cytoplasmic staining was achieved by incubating the samples in eosin solution for 2 min, followed by similar washing steps. The stained sections were then examined under a light microscope (Diaphot 200, Nikon Corporation, Tokyo, Japan).

### Statistical analysis

All experiments were performed in triplicate (n = 3 independent samples per group) unless otherwise stated. Data are reported as mean ± standard deviation (SD). For time-course data (Days 1, 7, and 14), two-way ANOVA (factors: group and time) followed by Tukey’s multiple-comparisons test was applied; single-time-point comparisons used one-way ANOVA with Tukey’s test. P values were computed using standard statistical software, and significance was accepted at p < 0.05 (two-tailed). Exact p-values are reported when possible.

## Results and discussions

### Macroscopic evaluation and parameter justification

**Fig. 2:**
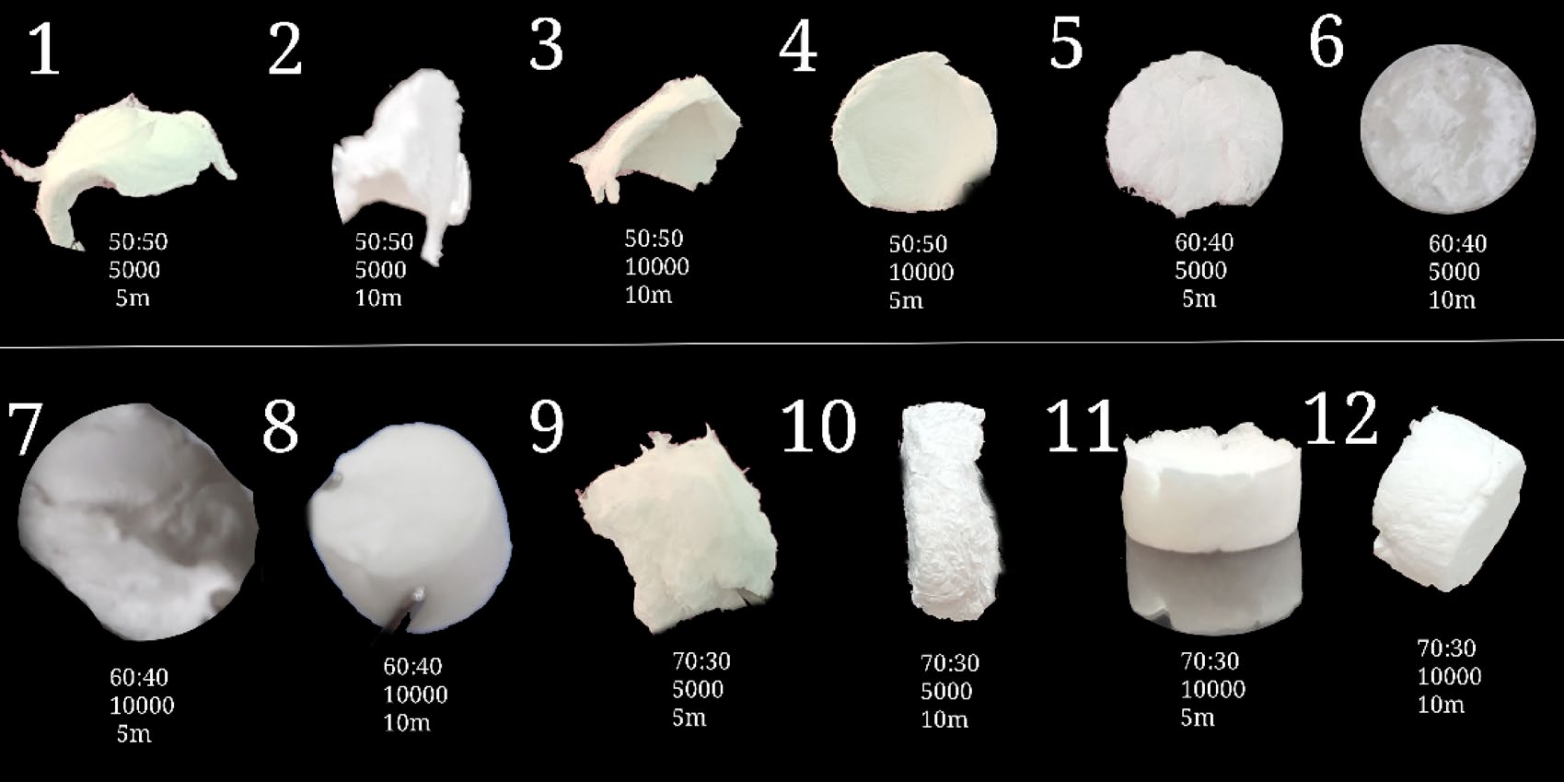
Macroscopic morphology of fabricated PCL/gelatin scaffolds with varying polymer ratios (50:50, 60:40, and 70:30) and centrifugal conditions (5000 and 10,000 rpm for 5 and 10 min).

The morphological appearance of all fabricated scaffolds is shown in Fig. 2, corresponding to the processing parameters listed in Table 2. Macroscopic evaluation revealed consistent and substantial differences between scaffolds centrifuged at 5000 rpm versus those at 10,000 rpm, independent of the polymer blend ratio.

Scaffolds fabricated at 5000 rpm exhibited severe structural imperfections, including irregular and asymmetric shapes, poor volume retention, surface wrinkling, and in some cases, partial collapse. These samples (Fig. 2, samples 1, 2, 5, 6) lacked coherent cylindrical geometry and showed signs of heterogeneous fiber accumulation, likely due to insufficient gravitational compaction during spinning. This irregularity not only compromises their resemblance to clinically usable scaffold architectures but also suggests limited reproducibility and poor process control under lower centrifugal conditions.

In contrast, the 10,000 rpm scaffolds (Fig. 2, samples 7, 8, 11, 12) consistently demonstrated superior structural attributes, including uniform, dense, and symmetrical cylindrical forms with smooth surfaces and robust macro-architecture. These morphological improvements can be directly attributed to the higher centrifugal force applied during the shaping phase, which likely facilitated enhanced fiber alignment and denser packing at the bottom of the coagulation bath^37^. Moreover, increased rpm may improve fluid removal and scaffold solidification, leading to sharper boundaries and better-defined 3D integrity^38^.

While macroscopic shape alone is not a sufficient criterion for complete scaffold evaluation, it represents a critical early-stage quality control parameter. Structural stability and geometric consistency are essential prerequisites for downstream processes such as cryosectioning, mechanical compression testing, and in vitro cell seeding. In our preliminary handling trials, scaffolds from the 5000-rpm group were significantly more fragile, prone to deformation under minimal manipulation, and unable to retain consistent dimensions after freeze-drying. These observations were further supported by poor visual reproducibility between replicates, indicating batch inconsistency under lower rpm.

For these reasons, and to preserve the scientific integrity and reliability of subsequent characterization, all samples fabricated at 5000 rpm were excluded from further analysis (including SEM, porosity, mechanical properties, and biological evaluations). This exclusion is not based solely on visual appearance but is supported by a combination of structural instability, handling difficulty, and lack of dimensional reproducibility, all of which undermine their potential utility in tissue engineering contexts. The study thus focuses exclusively on scaffolds produced under optimized 10,000 rpm conditions, which reliably yielded constructs with morphology, handling quality, and geometric fidelity compatible with both experimental testing and biomedical application.

To ensure the selection of practical and reproducible fabrication conditions, the following considerations were taken into account regarding the centrifugal speed and duration:

The selection of centrifugal speeds (5000 and 10,000 rpm) and durations (5 and 10 min) was informed by the physicochemical behavior of the polymeric system and the dynamic interactions occurring during wet electrospinning. The PCL/gelatin solution used in this study exhibited moderate to high viscosity, and preliminary assessments indicated that speeds below 5000 rpm failed to generate sufficient centrifugal force to compact the nanofibers within the coagulation bath^39^. As a result, these conditions led to loosely assembled or irregularly shaped scaffolds that lacked structural integrity. Conversely, centrifugal speeds above 10,000 rpm induced excessive compaction and fiber distortion, likely due to shear-induced instability, bath turbulence, and uncontrolled fluid dynamics, which compromised scaffold morphology and uniformity. In terms of centrifugation duration, times shorter than 5 min were insufficient to support fiber entanglement and three-dimensional shape stabilization^19^, while durations beyond 10 min caused over-densification of the scaffold and reduced porosity, which is undesirable for nutrient diffusion and cell migration. Furthermore, extended exposure of gelatin to the aqueous environment in the coagulation bath may promote premature swelling or partial degradation of the biopolymer component^40^. Taken together, the selected range of 5,000–10,000 rpm for 5–10 min represents a theoretically and practically optimized window for achieving mechanically stable, morphologically uniform, and biologically compatible 3D scaffolds. These constraints are dictated by the interplay between centrifugal force, polymer viscosity, solvent exchange, and scaffold architecture formation, and thus provide a rational framework for parameter selection in this system.

### SEM examinations of scaffolds

The main distinction between conventional dry and wet electrospinning processes lies in the ability of the wet process to fabricate 3D nanofibrous scaffolds^41,42^. In this study, the packing density and fiber distribution were evaluated for scaffolds with PCL-to-gelatin ratios of 50:50, 60:40, and 70:30. Various tests were performed to identify the optimal composition for cell applications.

**Fig. 3:**
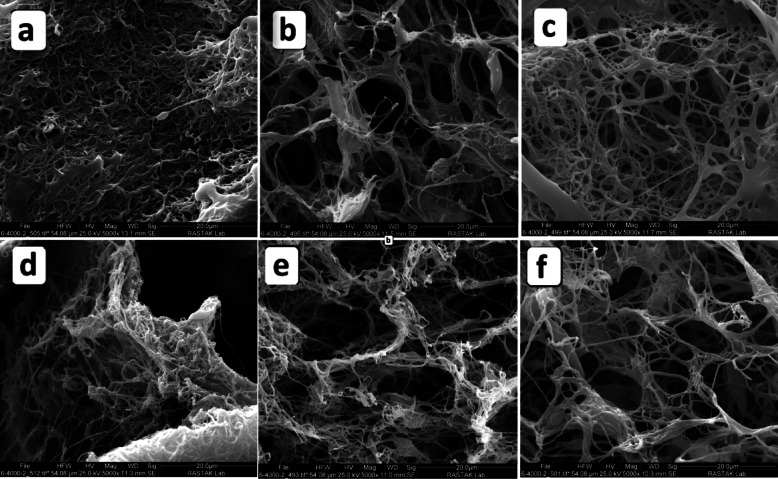
SEM micrographs of PCL/gelatin scaffolds with different ratios (**a**, **d**: 50:50; **b**, **e**: 60:40; **c**, **f**: 70:30) fabricated at 10,000 rpm for 5 min. Surface views (**a**–**c**) and cross-sections (**d**–**f**) reveal distinct morphological differences.

Figure 3 presents SEM micrographs of the surface and cross-sectional morphologies of scaffolds with different PCL/gelatin ratios (50:50, 60:40, and 70:30), all fabricated under the same conditions (10,000 rpm, 5 min). These images reveal distinct differences in fiber architecture, porosity, and internal connectivity across the formulations.

In the surface view of the 50:50 scaffold (Fig. 3a), the morphology appears highly compact and densely packed, with indistinct fiber boundaries and minimal visible porosity. This excessive surface compaction may hinder cell attachment and nutrient diffusion. The corresponding cross-sectional image (Fig. 3d) further confirms the absence of internal porosity, revealing a dense, almost solid structure with limited fiber interconnectivity. This suggests poor potential for deep cell infiltration and nutrient transport, both of which are essential for effective tissue regeneration.

The 60:40 scaffold (Fig. 3b**, e**) exhibits a well-defined fibrous network with a balanced distribution of pores at both the surface and cross-sectional levels. In Fig. 3b, the surface presents clearly distinguishable, interconnected fibers and moderately sized open pores. The cross-sectional view (Fig. 3e) reveals a three-dimensional porous network with uniform fiber layering and continuous pathways for nutrient exchange. These morphological features closely resemble the ECM, which supports cellular adhesion, migration, and metabolic function^43^.

In contrast, the 70:30 scaffold shows less favorable characteristics. The surface image (Fig. 3c) reveals an irregular fibrous structure with thinner and sparsely distributed fibers, leading to non-uniform porosity. The cross-sectional view (Fig. 3f) demonstrates high porosity, yet the structure appears disorganized, with heterogeneous fiber distribution and weak architectural cohesion. Such irregularity may compromise mechanical stability and reduce scaffold reproducibility.

Collectively, these observations indicate that the 60:40 PCL/gelatin scaffold offers the most desirable morphology for tissue engineering applications. It demonstrates a balance between porosity, fiber interconnectivity, and structural integrity, while the 50:50 scaffold is overly compact and lacks adequate pore structure. The 70:30 formulation, although highly porous, exhibits structural inconsistency and may be mechanically unstable.

**Fig. 4:**
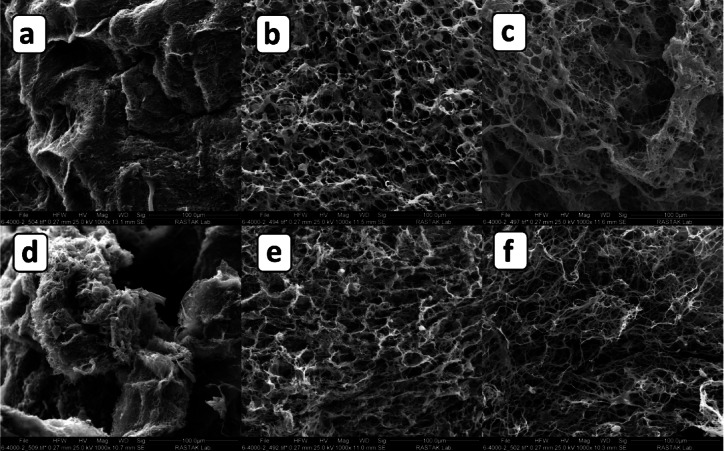
SEM micrographs of PCL/gelatin scaffolds at 10,000 rpm and different ratios: surface views of (**a**) 50:50 (10 min), (**b**) 60:40 (10 min), and (**c**) 70:30 (10 min); cross-sectional views of (**d**) 50:50 (10 min), (**e**) 60:40 (10 min), and (**f**) 70:30 (10 min).

To further validate and quantify the observed morphological differences, higher-magnification SEM imaging was performed (Fig. 4), enabling a more detailed examination of fiber organization and internal scaffold architecture. As shown in Fig. 4, distinct differences in morphology were observed among scaffolds with varying PCL/gelatin ratios. The 50:50 scaffold (Fig. 4a) exhibited a compact surface with poorly distinguishable nanofibers and minimal visible porosity. This may be attributed to the higher gelatin content, which can disrupt stable fiber elongation during electrospinning and lead to premature fiber fusion. The cross-sectional view (Fig. 4d) further confirmed the presence of a dense, non-porous core lacking internal fiber alignment or 3D interconnectivity, which is suboptimal for cell infiltration and nutrient transport.

Although the 50:50 scaffold appeared dense and visually non-porous in SEM images (Fig. 4a, d), its porosity was calculated using the ethanol displacement method based on Archimedes’ principle, as described in the methods section. This approach quantifies sub-surface microvoids not easily visible in surface SEM imaging.

In contrast, the 60:40 scaffold (Fig. 4b) demonstrated a well-organized surface network with evenly distributed pores and distinct nanofibers. Its cross-section (Fig. 4e) revealed a moderately porous internal architecture with interconnected fibers, indicating a favorable balance between mechanical integrity and permeability. The 70:30 scaffold (Fig. 4c, f) showed an open surface structure with large pores but a more heterogeneous internal morphology. While it achieved high porosity, inconsistencies in fiber packing and alignment could compromise its mechanical stability and structural reproducibility.

**Fig. 5:**
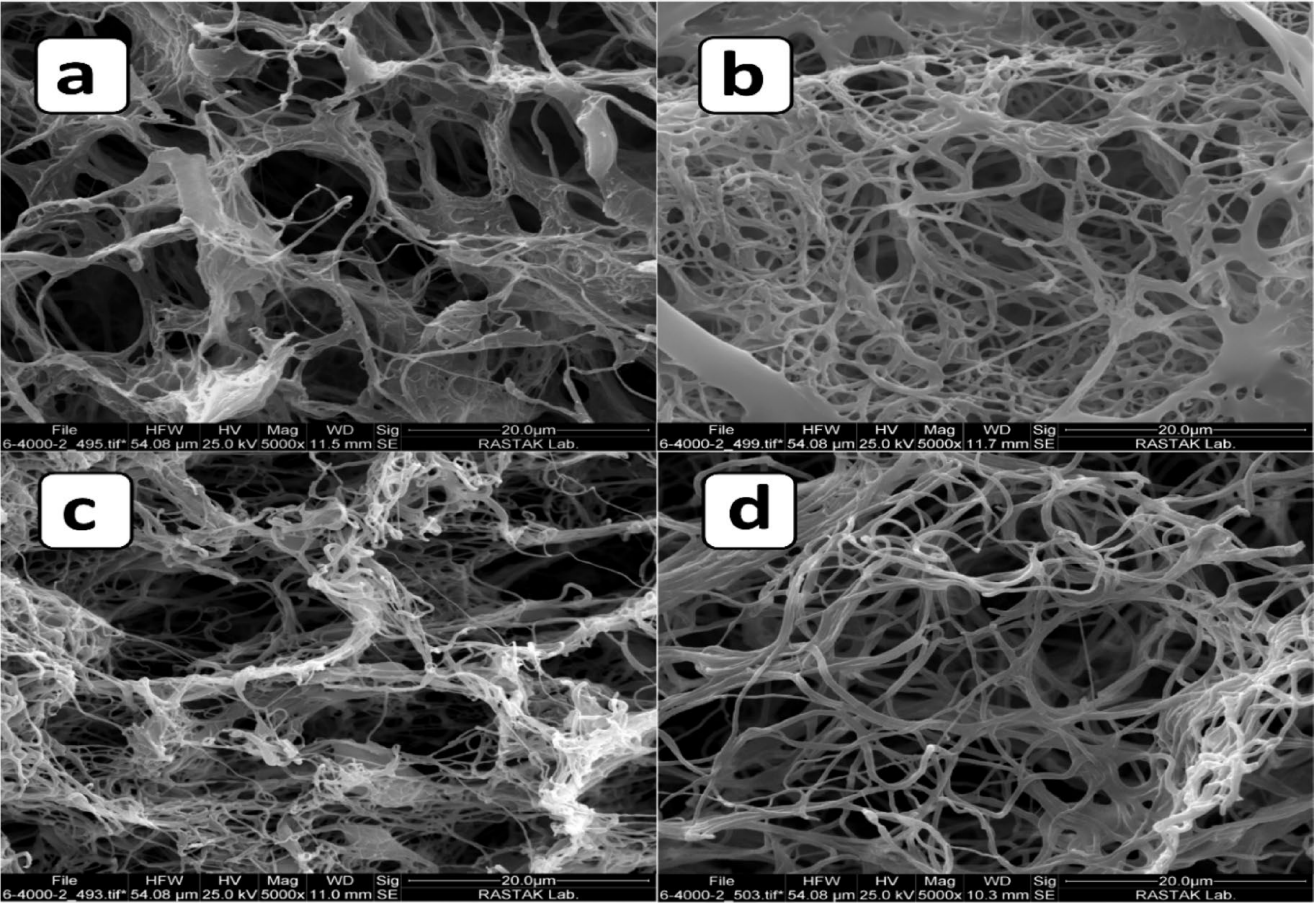
A higher magnification of SEM morphological evaluations of PCL/gelatin surface at 10,000 rpm with the ratio of (**a**) 60:40, 10 min, (**b**) 70:30, 10 min, and cross-sectioned PCL/gelatin with the ratio of (**c**) 60:40, 10 min, and (**d**) 70:30, 10 min.

In addition to qualitative morphological observations, quantitative analysis of fiber diameters was conducted using high-magnification SEM imaging (Fig. 5), providing insights into the microstructural characteristics of the scaffolds. As shown in Fig. 5, high-magnification SEM imaging was used to evaluate fiber morphology and quantify fiber diameters in both surface and cross-sectional views of the 60:40 and 70:30 PCL/gelatin scaffolds. The 60:40 scaffold exhibited average fiber diameters of 885 ± 156 nm on the surface (Fig. 5a) and 732 ± 102 nm in the cross-section (Fig. 5c), while the 70:30 scaffold displayed thicker fibers with diameters of 1015 ± 119 nm (Fig. 5b) and 1192 ± 94 nm (Fig. 5d), respectively.

This increase in fiber thickness with higher PCL content is attributed to the reduced electrical conductivity and increased viscosity of the solution, which limits jet stretching during electrospinning^44^. Notably, the smaller standard deviation values in cross-sectional measurements suggest a more uniform fiber distribution within the scaffold interior. Both formulations yielded uniform, bead-free nanofibers, with interconnected porous networks favorable for cell attachment and nutrient transport^45^. These nanoscale and microscale structural features closely resemble those of native ECM, thereby enhancing the scaffold’s suitability for tissue engineering applications^46^.

Based on the comparative SEM analyses presented in Figs. 3, 4, and 5, the morphological features of scaffolds with different PCL/gelatin ratios (50:50, 60:40, and 70:30) were systematically evaluated. The 50:50 scaffold consistently demonstrated suboptimal characteristics across all visual assessments. Its surface and cross-sectional images revealed excessively compacted structures with minimal visible porosity and indistinct fiber architecture, likely resulting from the high gelatin content that compromises fiber elongation and structural separation during electrospinning. Moreover, the lack of a discernible three-dimensional fibrous network and the solid, non-permeable interior structure (Figs. 3a, d and 4a, d) suggest poor potential for nutrient diffusion, oxygen exchange, and cellular infiltration—essential properties for tissue engineering applications^47^. As such, this composition was excluded from further analysis.

In contrast, the 60:40 scaffold exhibited the most favorable morphological characteristics. It displayed a well-organized nanofibrous surface with uniform pores and a highly interconnected internal architecture (Figs. 3b, e and 4b, e), closely resembling native ECM. Additionally, high-magnification SEM images (Fig. 5a, c) confirmed the presence of bead-free, homogeneous fibers with moderate diameters (885 ± 156 nm surface; 732 ± 102 nm cross-section), which offer a balance between mechanical support and surface area for cellular adhesion. The consistent standard deviation values also indicate excellent fiber uniformity, particularly within the scaffold core.

Although the 70:30 scaffold exhibited high porosity and thicker fibers (Figs. 3c, f; 4c, f; 5b, d), its SEM structure was less uniform with some irregular fiber distribution and pore size. Therefore, based solely on morphological observations, the 60:40 formulation was initially considered to exhibit a superior balance of porosity, fiber morphology, and structural integrity. Nonetheless, both 60:40 and 70:30 scaffolds were comprehensively evaluated in mechanical and biological assays.

### Porosity measurements of scaffolds

Porosity plays a crucial role in scaffold functionality, particularly for facilitating cell infiltration, nutrient exchange, and waste removal. Previous studies suggest that porosity values exceeding 80% are ideal for these functions^48,49^. However, higher porosity may compromise the mechanical stability of the scaffold, necessitating a balance between structure and function.

**Fig. 6:**
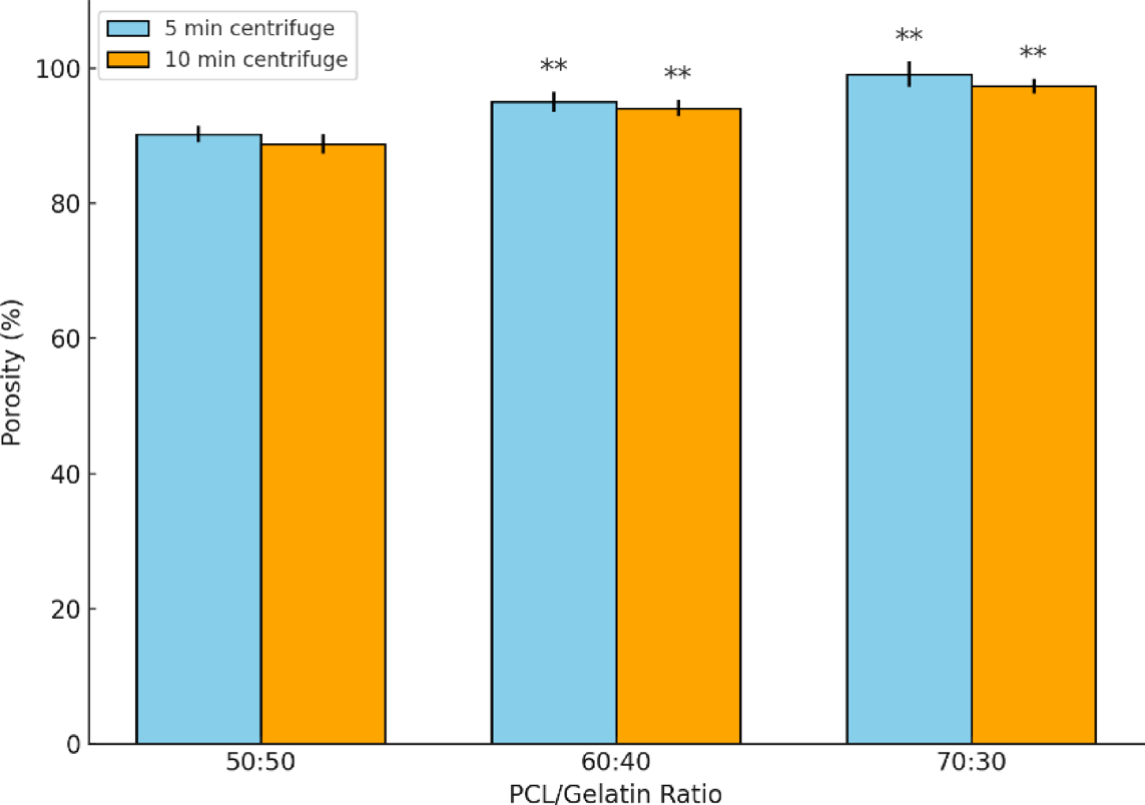
Porosity (%) of 50:50, 60:40, and 70:30 PCL/gelatin scaffolds at 5- and 10-min centrifugation at 10,000 rpm. Data shown as mean ± SD (n = 3). ** indicates significant difference (P < 0.05).

As shown in Fig. 6, significant differences in porosity were observed among the tested polymer compositions. The 60:40 and 70:30 scaffolds exhibited significantly higher porosity than the 50:50 formulation, regardless of centrifugation time. This trend is attributed to the higher PCL content in the 60:40 and 70:30 groups, which likely resulted in larger fiber diameters and wider inter-fiber spacing, promoting increased void volume. These findings align with prior studies reporting that greater fiber thickness and hydrophobicity of PCL can reduce fiber fusion and enhance pore formation^50,51^.

Notably, the centrifugation time (5 vs. 10 min) had no statistically significant effect on porosity within any given formulation. This suggests that the bulk porosity is primarily governed by fiber deposition dynamics during electrospinning, rather than post-collection compaction during centrifugation.

The highest porosity was measured in the 70:30 scaffold, reaching 98.1 ± 1.9% (5 min) and 97.3 ± 1.1% (10 min). The 60:40 scaffolds also exhibited very high porosity, 95 ± 1% (5 min) and 96 ± 1% (10 min). In contrast, the 50:50 group showed lower values, 90 ± 1% (5 min) and 89 ± 1% (10 min), consistent with its dense morphology observed in SEM images (Fig. 4a, d**)**.

Compared to other reported electrospun scaffolds, including CA/gelatin (75.83 ± 1.76%)^52^, dry-spun PCL/gelatin (65–75%)^53^, and others with porosities of 56.6%^54^ or 71.16%^55^, all formulations in this study showed markedly higher porosity. This highlights the effectiveness of wet electrospinning and centrifugal-assisted fiber deposition in producing highly porous matrices.

Furthermore, while 3D printed scaffolds typically achieve lower porosity (e.g., 79.32 ± 8.32%)^56^, the present wet electrospun systems outperformed them in both porosity and structural continuity. These results collectively underscore the advantages of optimized wet electrospinning for developing scaffolds with enhanced porosity, structural fidelity, and potential for improved cellular performance in tissue engineering applications.

These porosity results are also in agreement with the morphological findings obtained via SEM analysis. As demonstrated in Figs. 3 and 4, the 50:50 scaffold consistently exhibited a densely packed architecture, with minimal visible surface or internal porosity and limited fiber separation. This compact morphology is attributed to the high gelatin content, which reduces electrospinning jet elongation and promotes premature fiber fusion, ultimately preventing the formation of an open, interconnected nanofibrous network.

In addition to its visually non-porous structure, the 50:50 group also showed numerically the lowest porosity values (below 90%) among all tested compositions, as shown in Fig. 6. These findings collectively suggest that the 50:50 scaffold lacks the structural and porosity characteristics necessary to support efficient nutrient transport, cell migration, and tissue integration. Due to its poor morphological profile and inadequate porosity, the 50:50 composition was excluded from subsequent biological and mechanical assessments to focus on more promising formulations. This decision was also supported by its poor performance in SEM analysis and its incompatibility with the targeted biological application (neural tissue engineering), where open and interconnected porosity is critical for cell migration and nutrient diffusion.

### PBS uptake of scaffolds

**Fig. 7:**
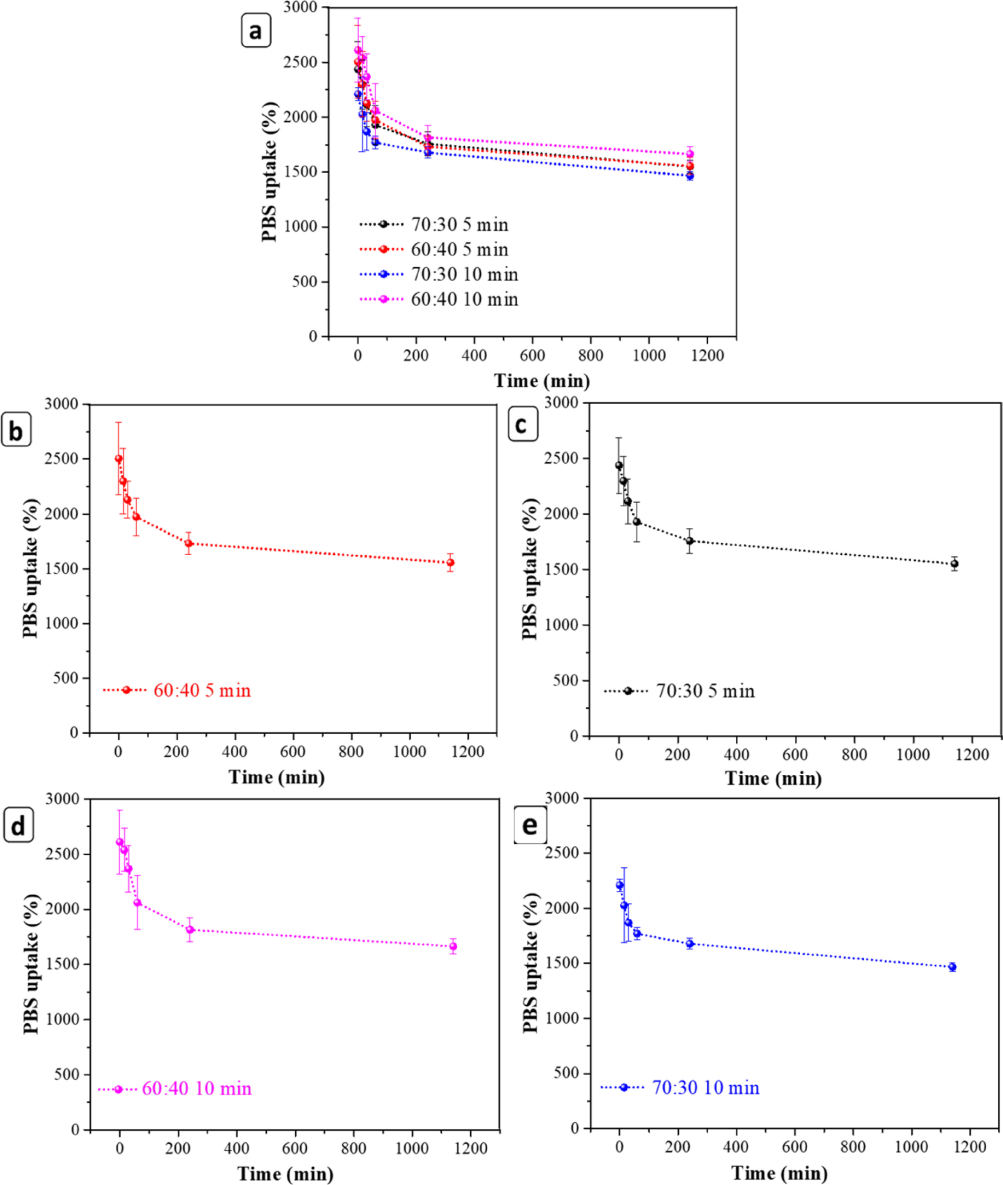
The PBS uptake of (**a**) all samples (**b**) 5 min 60:40 (**c**) 5 min 70:30 (**d**) 10 min 60:40 and (**e**) 10 min 70:30 PCL/gelatin scaffolds at 10,000 rpm. Data are presented as mean ± SD (n = 3).

The PBS absorption capacity is a key indicator of the hydrophilicity and fluid uptake potential of scaffolds, both of which are critical for supporting nutrient exchange and cellular adhesion in tissue engineering applications^57^. To assess this property, scaffolds with different PCL-to-gelatin ratios (60:40 and 70:30) and centrifugation durations (5 and 10 min) were immersed in PBS, and the uptake (%) was measured over a 24-h period, as shown in Fig. 7.

The maximum PBS absorption values followed the order: 60:40–10 min > 60:40–5 min > 70:30–5 min > 70:30–10 min. This trend underscores the combined effects of polymer composition and centrifugation time on the hydrophilic performance of the scaffolds. Visual readings from the plots indicate that at 24 h the equilibrium PBS uptake (Q_∞_) was approximately 40 ± 1 percent for 60:40 for 10 min, 36 ± 1 percent for 60:40 for 5 min, 33 ± 1 percent for 70:30 for 5 min, and 30 ± 1 percent for 70:30 for 10 min. In the early phase within the first hour, the 60:40 for 10 min scaffold also showed the fastest rise, reaching about 55 ± 2 percent at 1 h compared with 52 ± 2 percent for 60:40 for 5 min, 49 ± 2 percent for 70:30 for 5 min, and 45 ± 2 percent for 70:30 for 10 min. At 15 min, the initial uptake followed the same ranking, with approximate values of 80 ± 2 percent, 78 ± 2 percent, 73 ± 2 percent, and 68 ± 2 percent for the four groups respectively.

Scaffolds with a lower PCL content (60:40) consistently exhibited higher PBS uptake than those with a higher PCL content (70:30). This is attributed to the inherent hydrophobicity of PCL, which limits water absorption^58^, whereas gelatin, being rich in polar amino acid residues with amine and carboxylic groups, readily forms hydrogen bonds with water molecules^59^. This behavior is consistent with previous studies on other hydrophilic biopolymers such as chitosan^60^.

Within the 70:30 group, scaffolds centrifuged for only 5 min exhibited slightly higher PBS absorption than those processed for 10 min. This difference can be attributed to the lower degree of fiber compaction in the 5-min group, which preserves greater residual pore volume and enhances fluid diffusion. In contrast, prolonged centrifugation promotes tighter fiber packing and scaffold densification, which may inadvertently reduce the accessible porosity and hinder PBS penetration—particularly in PCL-rich systems where the intrinsic hydrophobicity limits capillary-driven fluid uptake. These findings suggest that in polymer compositions with higher PCL content, shorter centrifugation durations may better preserve the microstructural openness required for efficient fluid interaction, thus supporting enhanced hydration and potentially better biological integration^61^. For the 60:40 group, increasing the centrifugation time from 5 to 10 min improved PBS uptake, likely due to enhanced inter-fiber connectivity and improved scaffold consolidation. These effects enabled more efficient hydration and fluid retention without significantly compromising overall porosity.

The PBS absorption profiles displayed a sharp initial uptake within the first hour, attributed to the high surface area and open nanofibrous architecture, followed by a plateau phase as the scaffolds approached saturation^62^. Notably, the 60:40–10 min condition exhibited the highest overall PBS absorption and the most sustained retention across the full 24-h period, highlighting it as the optimal formulation for hydrophilic and diffusion-permissive scaffold design. Among the tested conditions, the scaffold with 60:40 polymer ratio and 10-min centrifugation demonstrated the best balance of porosity, hydrophilicity, and fluid retention capacity, making it the most promising candidate for applications requiring enhanced nutrient and metabolite exchange.

### Mechanical properties of scaffolds

**Fig. 8:**
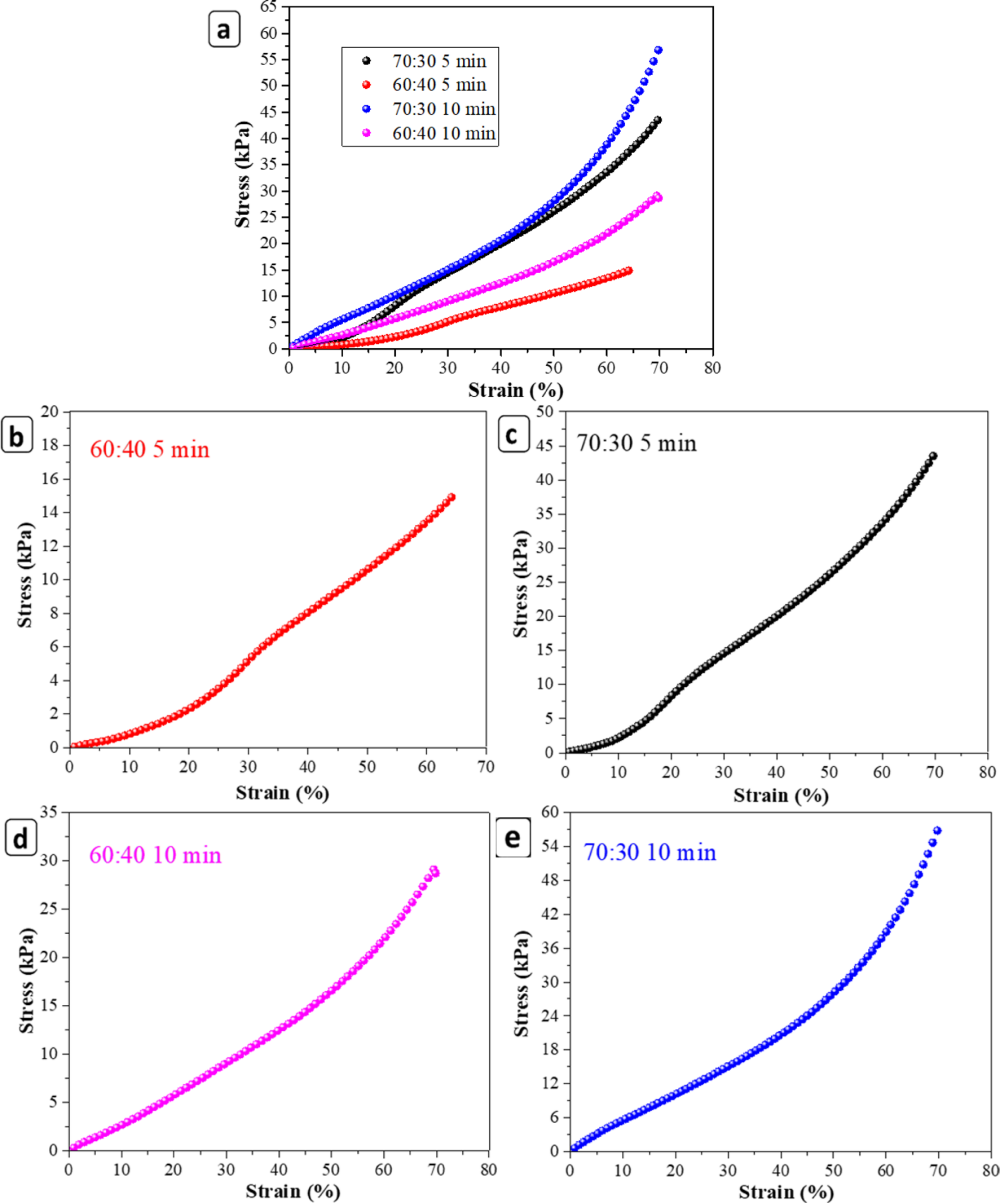
The compressive behavior of (**a**) 5 min 60:40 (**b**) 5 min 70:30 (**c**) 10 min 60:40 and (**d**) 10 min70:30 PCL/gelatin scaffolds at 10,000 rpm. Data are presented as mean ± SD (n = 3).

**Fig. 9:**
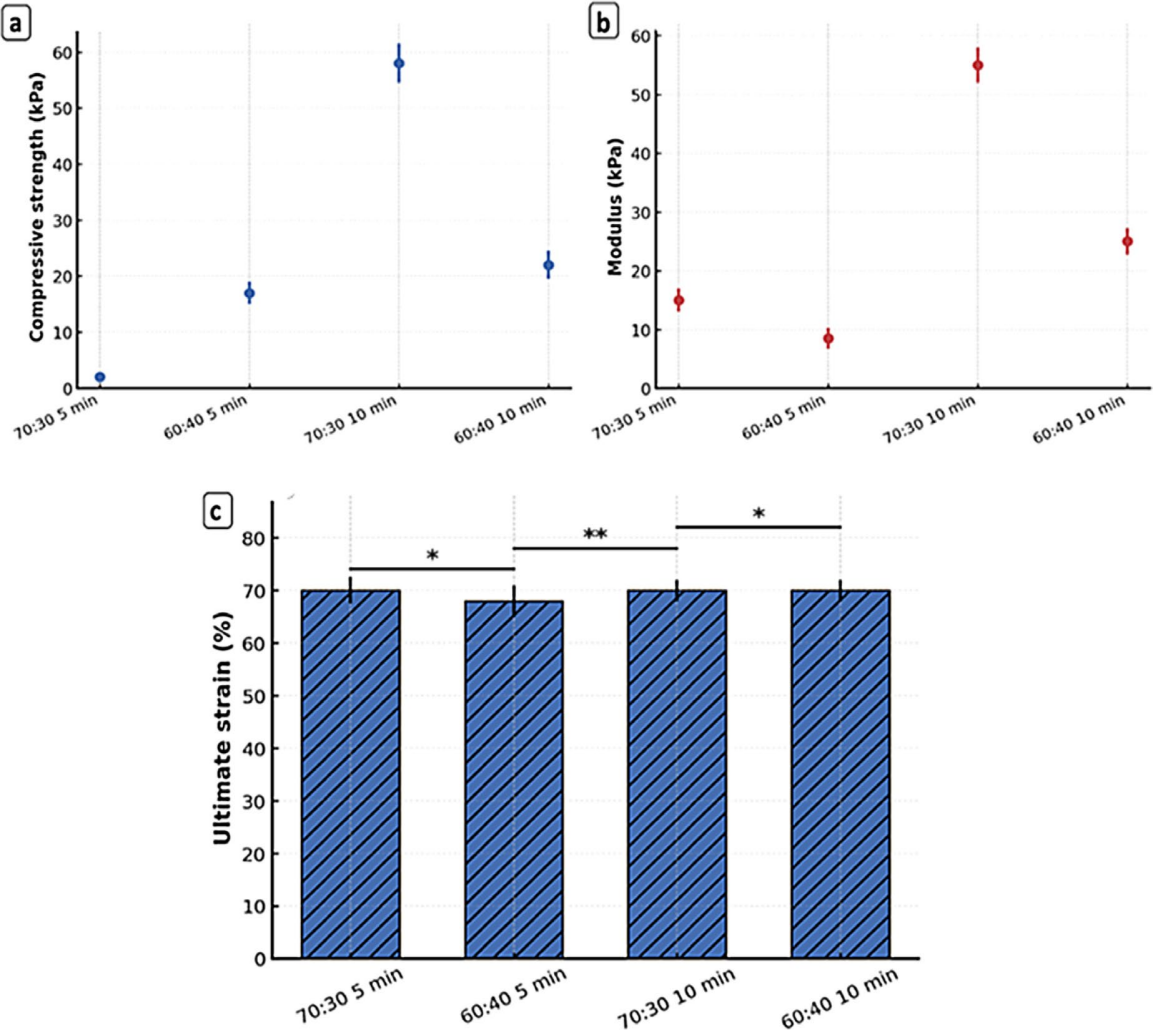
(**a**) Compressive strength of 3D PCL/gelatin scaffolds (60:40 and 70:30) fabricated with 5- and 10-min centrifugation; (**b**) Corresponding compressive modulus for each formulation; (**c**) Ultimate strain (%) of scaffolds. Data are presented as mean ± SD (n = 3). Significant differences between groups are indicated (*p < 0.05, **p < 0.01).

Figures 8 and 9 illustrate the representative compressive stress–strain curves for scaffolds fabricated with two polymer blend ratios (60:40 and 70:30 PCL/gelatin) and two centrifugation durations (5 and 10 min). The corresponding quantitative mechanical parameters, including compressive strength, compressive modulus, ultimate compressive strain, maximum load, and deformation at failure, are summarized in Table 3.

**Table 3:** The parameters of mechanical assessments are summarized.

Sample	Compressive strength (kPa)	Compressive modulus (kPa)	Ultimate compressive strain (%)	Maximum force (N)	Elongation (mm)
70:30 5 min	43.74 ± 2.00	21.00 ± 1.00	69.95 ± 0.38	5.80 ± 0.26	5.61 ± 0.03
60:40 5 min	17.69 ± 3.75	10.40 ± 3.70	67.90 ± 3.38	2.72 ± 0.46	4.74 ± 0.21
70:30 10 min	57.03 ± 1.50	53.00 ± 2.00	70.08 ± 0.36	8.77 ± 0.23	4.45 ± 0.20
60:40 10 min	28.21 ± 1.38	24.70 ± 1.53	69.55 ± 0.35	4.34 ± 0.15	5.11 ± 0.03

Among all formulations, the 70:30 scaffold centrifuged for 10 min exhibited the most superior mechanical performance. It showed the highest compressive strength (57.03 ± 1.50 kPa), compressive modulus (53.00 ± 2.00 kPa), and maximum load (8.77 ± 0.23 N), while maintaining a high ultimate compressive strain (70.08%). This enhanced performance is primarily attributed to the higher PCL content, which provides robust semi-crystalline structural support, and the prolonged centrifugation time, which facilitates effective nanofiber compaction, alignment, and reduced inter-fiber voids^63^. The denser matrix resulting from longer centrifugation leads to stronger fiber–fiber interaction and more efficient load transfer^64^. These improvements are consistent with prior reports showing that increased polymer concentration or alignment enhances mechanical stability due to tighter chain entanglement and improved cohesion^65,66^.

In contrast, the 60:40 scaffold centrifuged for 5 min displayed the weakest mechanical properties, with the lowest compressive strength (17.69 ± 3.75 kPa) and modulus (10.40 ± 3.70 kPa). These limitations arise from both the higher gelatin content—an inherently brittle, hydrophilic polymer—and insufficient gravitational compaction, leading to heterogeneous and loosely connected fiber structures^67^.

Although ultimate compressive strain values (70%) were comparable across all groups, the deformation at failure (mm) differed. The 70:30 scaffold centrifuged for 5 min exhibited the highest deformation (5.61 mm), indicating greater capacity for structural compression before failure. This suggests that shorter centrifugation preserves a more compliant structure, allowing higher compressive displacement under load. However, this flexibility comes at the expense of reduced compressive strength and stiffness. Such trade-offs between compliance and rigidity are crucial in tailoring scaffold properties to specific tissue engineering needs^68^.

From a biofunctional perspective, the 70:30–10 min scaffold provides an optimal balance of stiffness and strength, with a compressive modulus (53.00 ± 2.00 kPa) that falls within the physiological range reported for neural tissues, including peripheral nerves and dorsal root ganglia (10–140 kPa)^69^. Its high resistance to applied compressive loads and robust structural integrity make it a suitable candidate for applications requiring mechanical stability, such as nerve regeneration or musculoskeletal repair.

On the other hand, the 70:30–5 min scaffold presents an alternative advantage in applications where enhanced compressive deformability and flexibility are desirable, such as in softer or curved tissue regions. Despite having slightly lower compressive parameters, it retains sufficient strength and superior deformation behavior, which may support dynamic tissue interfaces more effectively.

In conclusion, both 70:30 scaffolds demonstrated favorable compressive performance, with the 10-min centrifugation variant being the most promising due to its strength, stiffness, and load-bearing capacity, while the 5-min variant offered greater deformability. These results underline the importance of precisely tuning polymer ratios and centrifugation parameters to engineer scaffolds that meet specific mechanical and biological requirements.

This 83% height reduction during centrifugation significantly contributed to the observed improvements in mechanical stability, as the denser fiber packing enhanced load transfer while maintaining open, interconnected porosity (> 97%). The compaction process thus provided a favorable balance between mechanical reinforcement and permeability, ensuring suitability for neural tissue engineering applications.

### Cellular morphology and biocompatibility of scaffolds

**Fig. 10:**
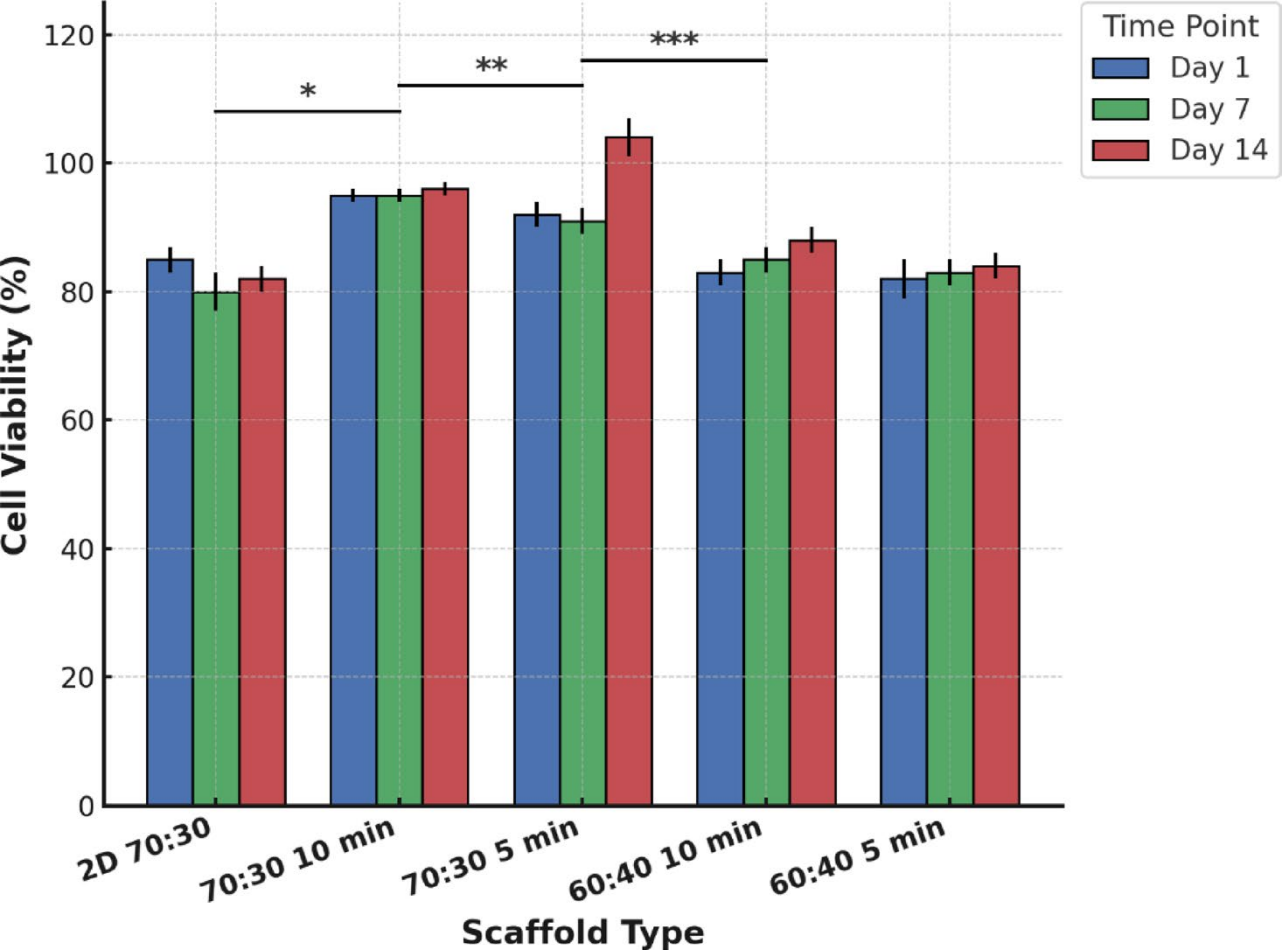
MTT assay results showing cell viability of C6 glial cells cultured on 2D 70:30 and 3D PCL/gelatin scaffolds (60:40 and 70:30) centrifuged for 5 and 10 min at 10,000 rpm, assessed after 1, 7, and 14 days. Data are presented as mean ± SD (n = 3). Significant differences between groups are indicated (*p < 0.05, **p < 0.01, ***p < 0.001).

Figure 10 presents the results of the MTT assay conducted to evaluate the biocompatibility and long-term proliferation of C6 glial cells cultured on different scaffold formulations over 14 days. The 2D 70:30 scaffold, used as the reference group, exhibited an initial cell viability of approximately 85% on day 1, which slightly decreased over time to around 80% on day 14. This declining trend is consistent with previous findings that 2D planar substrates impose spatial constraints on cell–cell and cell–matrix interactions, thereby limiting sustained proliferation^70^.

In contrast, all 3D scaffolds demonstrated superior and more dynamic proliferation profiles compared to the 2D control. Among the 70:30 scaffolds, both 5-min and 10-min centrifugation groups exhibited consistently high cell viability across all time points. The 70:30 scaffold centrifuged for 5 min showed the highest viability at day 14, surpassing even the 10-min variant. Specifically, the 70:30–5 min group achieved viability levels exceeding 100% at day 14 (+ 12.16%), indicating robust and sustained metabolic activity and proliferation. This exceptional performance is likely driven by the optimal balance of mechanical properties and preserved porosity, which together support enhanced nutrient diffusion, oxygen transport, and favorable mechanotransduction^71^. Moreover, this elevated metabolic activity may also reflect enhanced mitochondrial function and ECM remodeling, processes that are typically promoted in well-structured, highly porous 3D environments^72^.

The 70:30 scaffold centrifuged for 10 min also maintained high viability across all time points, though its day 14 viability was slightly lower than that of the 5-min group (+ 0.39% gain). This plateau in metabolic activity may result from excessive fiber compaction induced by prolonged centrifugation, which improves mechanical properties—as demonstrated in prior mechanical analyses—but may concurrently reduce effective pore openness and hinder the diffusion of oxygen and nutrients required for optimal proliferation^73^. Such a trade-off between scaffold mechanical integrity and mass transport capacity is a well-recognized phenomenon in dense electrospun scaffolds, particularly in neural tissue engineering applications.

For the 60:40 scaffolds, both 5-min and 10-min groups exhibited lower overall viability compared to the 70:30 scaffolds but still outperformed the 2D control. The 60:40–10 min group showed a gradual increase in viability over time (+ 5.88%), indicating improved scaffold stability and microenvironmental support with extended centrifugation. In contrast, the 60:40–5 min scaffold maintained the lowest viability among all 3D groups (+ 1.01%), likely due to insufficient mechanical integrity and partial pore collapse, which impair sustained proliferation. The inherently lower bioactivity observed in 60:40 scaffolds can also be attributed to their higher gelatin content and lower PCL fraction, which collectively reduce scaffold stiffness and dimensional stability, thereby compromising the long-term biological performance^74^.

To design the subsequent morphological and histological evaluations (SEM, DAPI, H&E), scaffold selection was performed by considering both the results of the MTT assay and the complementary findings from mechanical and structural analyses. Although the 70:30 scaffold centrifuged for 5 min exhibited the highest relative increase in cell viability (+ 12.16%), its mechanical performance and structural uniformity were inferior to those of the 70:30 10-min scaffold, which demonstrated superior tensile strength, modulus, and optimal porosity in prior assessments. Given that neural tissue engineering applications require a balanced combination of biological performance and mechanical stability, the 70:30 10-min scaffold was selected as the representative PCL-rich formulation for further cellular characterization. Moreover, within the 60:40 group, the scaffold centrifuged for 10 min consistently outperformed the 5-min variant across both MTT and mechanical tests, justifying the exclusion of the 5-min scaffold from subsequent analyses. Accordingly, only the 10-min scaffolds for both 70:30 and 60:40 compositions were included in the following SEM, DAPI, and H&E staining studies. This selection strategy ensured that comparative cellular and morphological evaluations were conducted on scaffolds exhibiting the most favorable combination of mechanical integrity, structural properties, and biological performance, thereby providing a comprehensive and scientifically balanced assessment of scaffold suitability. The absence of chemical crosslinking did not compromise scaffold stability during the entire experimental period. This stability was attributed to the dense nanofibrous architecture formed by centrifugal wet electrospinning, where PCL provided mechanical reinforcement and minimized gelatin dissolution. The scaffolds maintained their morphology during PBS uptake assays, repeated medium exchanges, and 14-day cell cultures, confirming that physical reinforcement by PCL was sufficient to retain structural integrity without additional crosslinking.

**Fig. 11:**
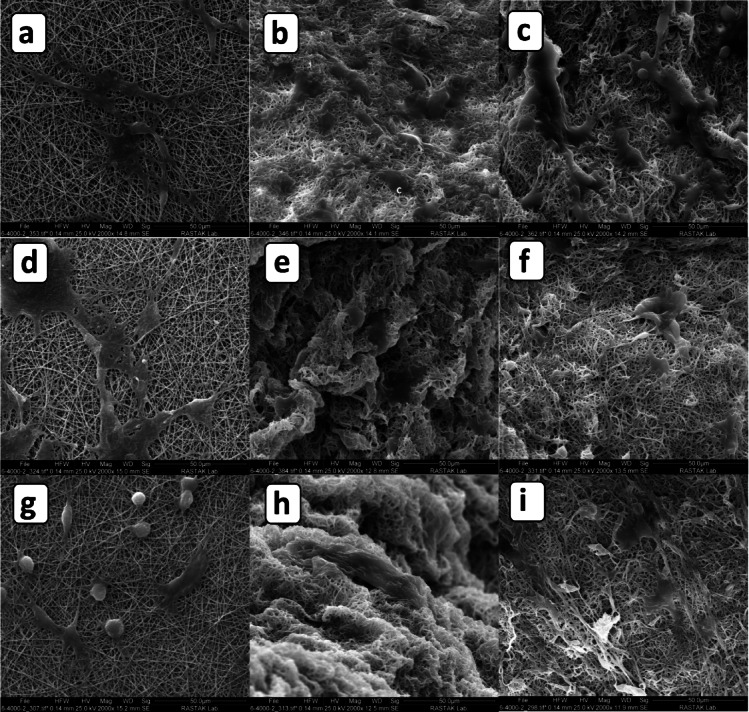
SEM images of C6 glial cells cultured on PCL/gelatin scaffolds after 1 day (**a**–**c**), 7 days (**d**–**f**), and 14 days (**g**–**i**). For each time point: (**a**, **d**, **g**) show 2D 70:30; (**b**, **e**, **h**) show 3D 60:40; and (**c**, **f**, **i**) show 3D 70:30 scaffolds. All 3D scaffolds were fabricated with 10-min centrifugation.

SEM imaging was conducted on days 1, 7, and 14 to evaluate the morphological evolution of C6 glial cells cultured on 2D and 3D PCL/gelatin scaffolds (Fig. 11). The 3D scaffolds (60:40 and 70:30 ratios) were fabricated using 10-min centrifugation to ensure consistent fiber compaction and structural integrity.

On day 1, cells on the 2D 70:30 scaffold (Fig. 11a) exhibited flat, well-spread morphologies characteristic of substrate-adherent cells. In contrast, those on 3D scaffolds (Fig. 11b, c) showed more rounded morphologies with limited spreading, indicative of early adaptation within a three-dimensional matrix. Over time, cells on 3D scaffolds—particularly the 70:30 group—underwent elongation and acquired spindle-shaped morphologies with distinct dendritic extensions (Fig. 11f, i), reflecting neural-like behavior and enhanced substrate interaction.

This morphological transformation was less pronounced in the 60:40 scaffold (Fig. 11e, h), where cells remained more clustered with reduced spreading, possibly due to microenvironmental instability arising from gelatin-rich composition^75^. The 70:30 scaffold provided a more favorable mechanical and architectural niche, mimicking ECM properties such as stiffness and fiber orientation. These characteristics are essential for neural phenotype maintenance and have been shown to promote cytoskeletal polarization and axon-like projection formation in prior studies^76,77^.

Importantly, 3D environments enable enhanced integrin engagement and cytoskeletal reorganization, supporting more physiologically relevant morphologies compared to 2D systems, which often trigger compensatory focal adhesion responses. The gradual transition of cells on 2D substrates—from fibroblastic shapes at day 1 to more elongated forms by day 14 (Fig. 11a, d, g)—further confirms cellular adaptation through surface-mediated tension rather than matrix-mediated signaling.

Overall, the SEM data underscore the superior performance of the 3D 70:30 scaffold in promoting neural-like morphology and cellular organization, likely due to a synergistic combination of mechanical stability, aligned nanofiber architecture, and reduced gelatin content that minimizes hydrolytic degradation during culture.

### Cellular staining methods of scaffolds

Nuclear staining is a definitive method to confirm cell organization and penetration within the 3D structure of scaffolds, offering qualitative insights that complement metabolic assays such as MTT, which may be influenced by polymer interference or optical artifacts. In this study, cell nuclei were visualized using DAPI staining to assess their spatial distribution, morphology, and interactions with the scaffold microstructure.

**Fig. 12:**
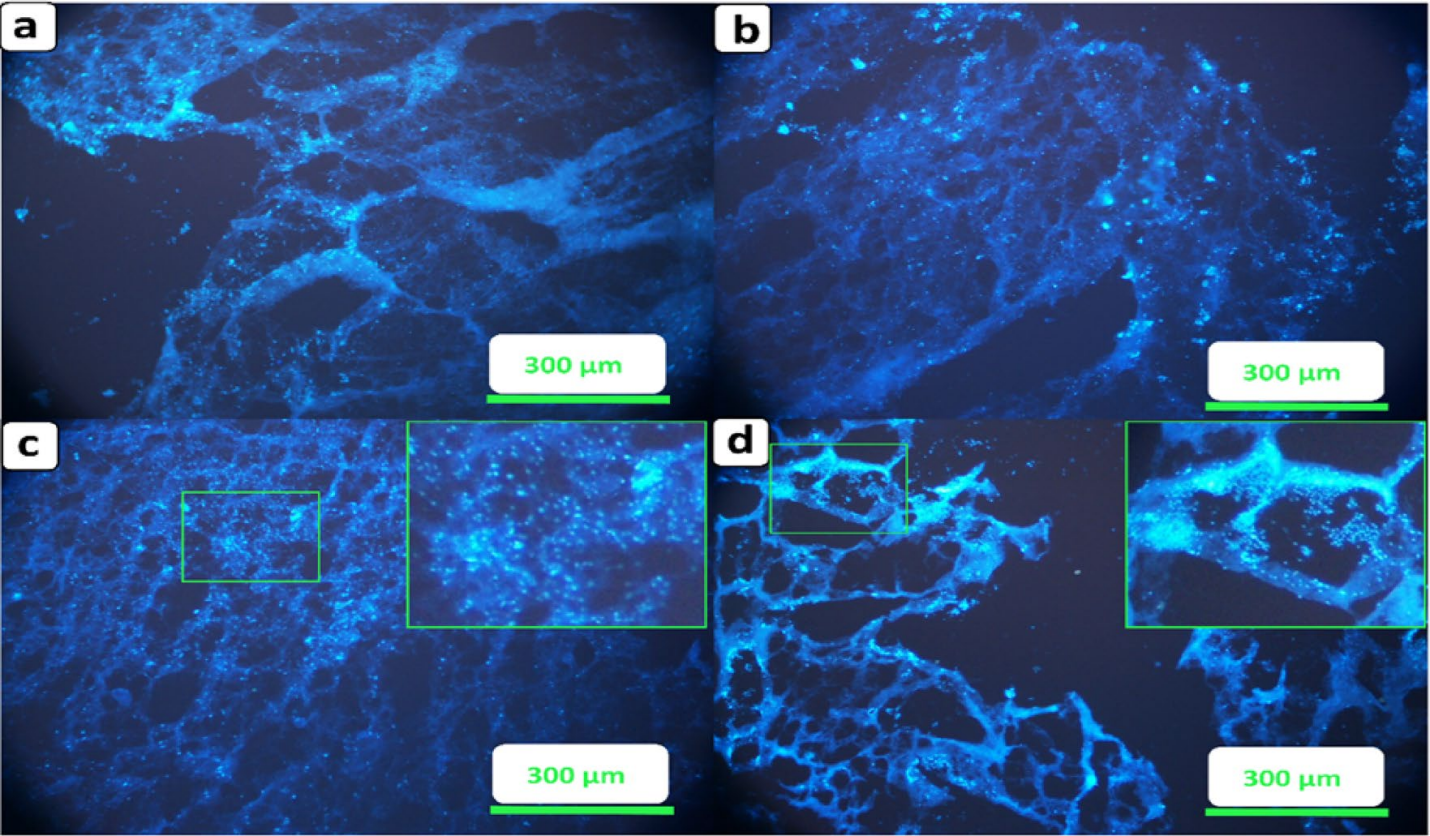
DAPI staining of cells after 7 days on (**a**) 60:40 (**b**) 70:30 and after 14 days on (**c**) 60:40 (**d**) 70:30. The squares are obtained by further magnifications to show cell aggregates.

As shown in Fig. 12, cells adhered throughout the scaffolds and exhibited clear nuclear staining across all groups. Higher cell densities and aggregation patterns were particularly evident in Fig. 12c, d, indicating enhanced cellular organization and clustering. These observations qualitatively support the MTT assay results, which showed increased metabolic activity over time.

Figure 12a, b showed markedly lower cellular density and no evidence of clustering or spatial organization. The 60:40 scaffold (Fig. 12a) exhibited weak nuclear signals with sparse, randomly distributed cells, indicating poor early-stage cell retention and interaction. Likewise, the 70:30 scaffold (Fig. 12b) showed slightly higher staining intensity but still lacked the pronounced cellular aggregation observed in later samples. These findings suggest that 7 days of culture were insufficient for establishing meaningful cell–scaffold interactions, particularly in gelatin-rich scaffolds with suboptimal structural support. Consequently, only the 14-day time points were considered for detailed morphological interpretation and scaffold comparison. Notably, the scaffold corresponding to the 70:30 formulation (Fig. 12d) demonstrated more pronounced cell aggregation and localized clustering compared to the 60:40 scaffold (Fig. 12c). This behavior is likely associated with improved structural stability and fiber compaction in the PCL-rich scaffold, which may better support sustained cell attachment and spatial guidance. PCL’s semi-crystalline nature and slower degradation profile provide a more stable substrate over extended culture durations, reducing matrix deformation and maintaining topographical integrity^78^. In contrast, the 60:40 scaffold showed lower overall cell density and fewer aggregation centers. This may be partially attributed to gradual gelatin release during medium exchange in the absence of crosslinking, leading to localized detachment of gelatin-adhering cells. This observation aligns with previous studies reporting that non-crosslinked gelatin in hybrid scaffolds can dissolve or swell over time, thereby compromising cell retention^79^. Moreover, gelatin’s hydrophilicity and water solubility can lead to softening of the scaffold matrix, which in turn reduces mechanical support for long-term cell anchorage^79^.

Overall, these nuclear staining results highlight the superior cellular compatibility and structural performance of the 70:30 scaffold, making it a promising candidate for 3D neural tissue culture models where scaffold integrity and sustained cell interactions are essential. These DAPI staining results are fully consistent with the MTT findings and SEM observations, reinforcing the suitability of the selected 10-min scaffolds (70:30 and 60:40) for further morphological and biological evaluation.

**Fig. 13:**
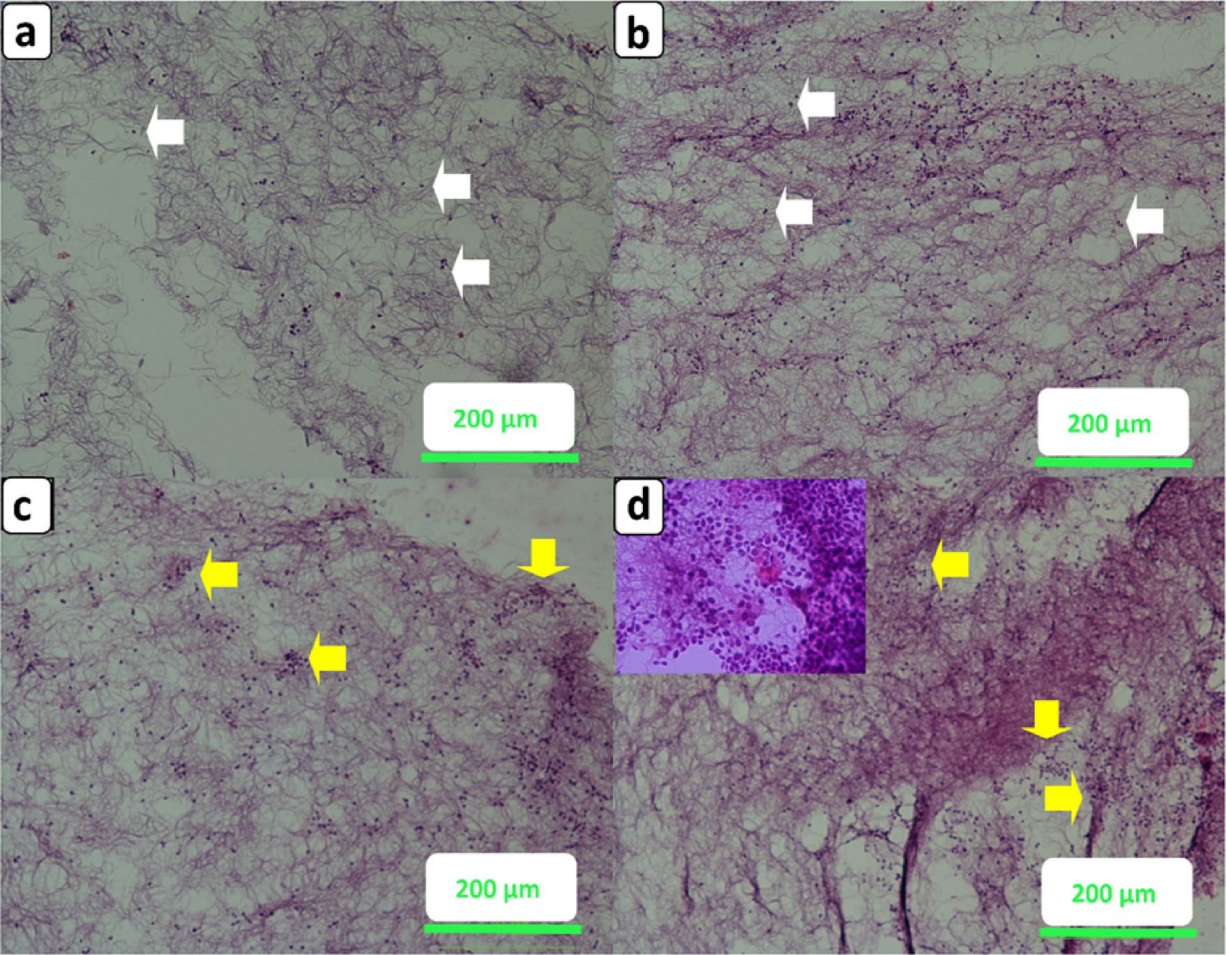
Hematoxylin–eosin staining of cells after 7 days on (**a**) 60:40 (**b**) 70:30 and after 14 days on (**c**) 60:40 (**d**) 70:30. The white and yellow arrows show single and aggregated cells respectively. The square reveals further magnifications to show cell aggregates.

H&E staining was used to evaluate the cellular morphology and distribution within the scaffolds after 7 and 14 days of culture (Fig. 13). At day 7, both the 60:40 (Fig. 13a) and 70:30 (Fig. 13b) scaffolds showed sparse cell populations, primarily present as isolated nuclei (white arrows) with limited evidence of aggregation. While the 70:30 scaffold exhibited slightly better cell retention compared to the 60:40, both groups lacked the well-defined cellular clusters necessary for robust tissue integration. These findings suggest that early cell-scaffold interactions were still in progress at this stage, particularly in the 60:40 scaffold where higher gelatin content may contribute to reduced mechanical support and partial cell detachment.

After 14 days, significant improvements in cell aggregation were observed. In the 60:40 scaffold (Fig. 13c), multiple small clusters (yellow arrows) were visible, though the density and size of aggregates remained modest. This limited organization may be attributed to gradual gelatin dissolution and matrix softening, which could compromise long-term scaffold integrity. In contrast, the 70:30 scaffold at day 14 (Fig. 13d) exhibited pronounced and dense cellular clusters, with multiple aggregation centers clearly visible.

The inset magnification in Fig. 13d reveals densely packed nuclei and eosinophilic regions, suggestive of active ECM deposition. Eosin stains basic proteins such as collagen and fibronectin, and its increased intensity in the 70:30 scaffold indicates robust ECM secretion by the adherent cells—an essential process for axonal guidance and neural repair^80^. These morphological patterns align with the previous DAPI and MTT results, collectively supporting the scaffold’s biocompatibility and ability to sustain neural-like cell behavior.

Although the 70:30 scaffold contains a lower proportion of gelatin, its superior dimensional stability appears to promote better long-term cell attachment and ECM retention. The semi-crystalline nature and slower degradation rate of PCL likely contribute to this outcome by minimizing scaffold collapse or gelatin dissolution during media exchanges. In contrast, the 60:40 scaffold may undergo partial matrix softening over time due to higher gelatin content, potentially limiting cell–matrix retention and aggregate stability.

Overall, these findings reinforce the importance of compositional tuning in scaffold design to ensure both biological responsiveness and structural endurance for neural tissue engineering applications. These H&E staining results are fully consistent with the previous MTT and DAPI findings, supporting the rationale for selecting the 10-min scaffolds (70:30 and 60:40) for detailed morphological and histological evaluations. This selection ensures a balanced assessment of scaffolds combining mechanical robustness with biological performance. Addressing the commonly cited limitation of highly porous 3D fibrous scaffolds, our data demonstrate that ultra-high volumetric porosity (> 97%) does not hinder cell attachment or proliferation when combined with neural-compatible kPa-range mechanics and preserved inter-fiber connectivity: C6 viability remained ~ 100–106% through Day 14 **(**Fig. 10), and DAPI/H&E/SEM confirmed deep cellular infiltration and ECM deposition, indicating authentic 3D integration rather than superficial colonization. Mechanistically, three features mitigate the classical trade-off: (i) tissue-matched compliance (≈25–53 kPa; 70:30, 10 min = 53.00 ± 2.00 kPa) supports focal-adhesion formation and productive mechanotransduction rather than the stress shielding seen with MPa-level mats; (ii) cell-adhesive, wettable fiber surfaces provided by gelatin (polar amino-acid residues) sustain hydration and protein adsorption, consistent with our 24 h PBS uptake (60:40, 10 min ≈ 40 ± 1% vs. 70:30, 10 min ≈ 30 ± 1%; Fig. 7); and (iii) a wet-collected, centrifugally compacted network maintains open, interconnected pores (porosity 97–98%; Fig. 6) and uniform fiber architecture (Figs. 3 and 4), shortening diffusion paths and preventing nutrient/oxygen limitations. Together, these factors decouple high porosity from loss of attachment, turning porosity into a transport advantage while mechanics and surface chemistry secure adhesion and proliferation.

## Discussion

Electrospun scaffolds are lauded for replicating ECM architecture, yet conventional dry electrospinning suffers from dense, laminar fiber mats with superficial porosity that severely limits three-dimensional cellular infiltration and nutrient transport (Table 4). Hwang et al.^74^ addressed this by incorporating crater like structures in PCL/gelatin fibers via gas foaming and salt leaching. Although this enhanced human mesenchymal stem cell proliferation and infiltration after 7 days, the reported porosity refers to crater area fractions rather than volumetric porosity, and mechanical integrity and spatial fiber connectivity remained poorly characterized.

**Table 4:** Summary comparsion.

Study	Key findings & strengths	Main limitations	Our scaffold improvements	Refs.
Hwang et al	Crater-like 2D mats (crater-area porosity 46–52%, superficial infiltration)	Low porosity, poor 3D structure, missing mechanical/cell data	> 97% volumetric porosity; true 3D nanofibrous structure; fully quantified	^74^
Bakhtiary et al	SPION-laden wet-spun scaffolds; improved PBS uptake and neural differentiation	No volumetric porosity or kPa mechanical data	Measured porosity, hydration, kPa-range mechanics, and long-term viability	^81^
Du et al	Enhanced strength, hydrophilicity, ROS scavenging, tissue repair in 2D mats	2D only; no 3D porosity or neural mechanics data	3D structure, > 97% porosity, hydration control, kPa mechanics, 3D integration	^82^
Perez–Puyana et al	Fiber alignment effects on 2D mechanics	Structural discontinuities, no porosity control, limited infiltration	Open 3D network, high porosity, mechanically tuned, improved cellular use	^83^

Bakhtiary et al.^81^ advanced the field by employing a magnetically assisted wet electrospinning process to generate PCL/gelatin scaffolds embedded with superparamagnetic iron oxide nanoparticles. Their study reported improved porosity, biodegradability, and PBS uptake, and supported enhanced neural differentiation. However, it lacked quantification of volumetric porosity, hydration dynamics at the same timepoints used here, mechanical properties in the kPa range, or systematic compositional optimization relevant to neural compatible mechanics.

Du et al.^82^ developed electrospun PCL/gelatin/arbutin nanofiber membranes demonstrating enhanced mechanical strength, increased hydrophilicity, improved water vapor transmission, and effective reactive oxygen species scavenging that accelerated wound healing via collagen deposition and epithelial regeneration in cutaneous models. While this membrane underscores how biochemical functionalization can enhance biological outcomes, it remains a two-dimensional construct, with no reported data on volumetric porosity, nutrient uptake kinetics at 24 h, or mechanical compliance within the kilopascal range. In contrast, our centrifugal wet electrospun PCL/gelatin scaffolds are engineered as true three-dimensional nanofibrous structures, offering volumetric porosity above 97 percent, tunable PBS uptake through compositional and centrifugal control, and a compressive modulus of 53 kPa tailored for neural tissue mechanics, along with validated long term glial infiltration and ECM formation. Thus, our design not only retains biochemical advantages but also addresses essential physical and functional requirements for neural tissue engineering that were not covered in the PCL/gelatin/arbutin system.

Perez Puyana et al.^83^ investigated fiber alignment effects on morphology and mechanics in PCL/gelatin scaffolds. They found that aligned fibers introduced structural discontinuities, whereas random fibers lacked directionality, and porosity remained uncontrolled. Their work did not explore a three-dimensional architecture or compatibility with neural specific mechanics.

In contrast, our centrifugal wet electrospun PCL/gelatin scaffolds integrate two complementary innovations. First, wet collection into a nonsolvent coagulation bath inherently produces a volumetric, interconnected, nanofibrous network rather than a dense two-dimensional mat. Second, a post deposition centrifugation compacts the fiber cloud vertically, achieving about 83 percent height reduction without collapsing the pore network, and preserving volumetric porosity above 97 percent as confirmed by ethanol displacement porosimetry.

A comprehensive evaluation of polymer ratios (50:50, 60:40, 70:30) under identical fabrication conditions revealed that the 50:50 scaffold was overly dense with poor fiber interconnectivity, while the 70:30 scaffold, though highly porous, lacked internal cohesion. The 60:40 scaffold consistently demonstrated an optimal balance, presenting bead free fibers, uniform architecture, and interconnected pathways akin to native ECM. SEM quantification showed average fiber diameters of 885 ± 156 nm on the surface and 732 ± 102 nm in cross section for the 60:40 scaffolds, while higher PCL content (70:30) led to thicker fibers, aligning with known effects of polymer viscosity on electrospinning jet stretching and fiber formation.

Hydration properties further underscore our design effectiveness. While gelatin increases hydrophilicity and PCL enhances structural stability, our PBS uptake ranked as follows: 60:40, 10 min > 60:40, 5 min > 70:30, 5 min > 70:30, 10 min. The improved uptake in the 60:40 scaffold with the longer centrifugation suggests optimized capillarity and inter fiber connectivity. Conversely, extended compaction in PCL rich scaffolds slightly reduces accessible voids, highlighting the importance of tuning centrifugation duration relative to composition.

Mechanical assessment places our scaffold within the physiological stiffness range of neural tissue. While many PCL based constructs exhibit MPa level moduli unsuitable for soft tissue, our optimized 70:30, 10 min scaffold achieves a compressive modulus of 53 kPa and compressive strength of 57 kPa, closely aligned with peripheral nerve mechanics in the kilopascal range.

Biological evaluations validate the functional advantages of our scaffold design. MTT assays reveal sustained C6 glial viability over 14 days, exceeding two dimensional controls. The 70:30, 5 min scaffold achieved the highest proliferation gain, while the 10 min variant delivered comparable biological performance with superior mechanical stability. DAPI and H and E staining further confirmed deep infiltration, clustering, and extracellular matrix deposition by day 14, indicating authentic three dimensional integration rather than superficial colonization.

In summary, our centrifugal wet electrospinning approach uniquely delivers ultra high volumetric porosity above 97 percent, tunable hydration behavior, neural compatible mechanics in the kPa range, and proven long-term three-dimensional cell viability and ECM formation. This integrated fabrication strategy robustly addresses major limitations of previous studies and provides a compelling, practice-oriented scaffold platform for neural tissue engineering. Unlike previous PCL/gelatin scaffolds that either enhanced biochemical properties without addressing volumetric architecture or improved porosity without achieving neural compatible mechanics, our platform combines ultra high volumetric porosity, optimal hydration kinetics, kPa range stiffness, and validated long-term three-dimensional cell viability in a single, fabrication ready system.

As summarized in Table 5, our three-dimensional PCL/gelatin scaffolds show ultra high volumetric porosity of 97 to 98 percent by ethanol displacement and neural compatible compressive moduli of about 25 to 53 kPa, together with controlled 24 h PBS uptake of about 30 to 40 percent and sustained C6 viability at day 14 of about 100 to 106 percent. Specifically, the 60:40, 10 min condition reached about 40 ± 1 percent PBS uptake at 24 h, while the 70:30, 10 min condition provided a compressive modulus of 53 ± 2 kPa. Both conditions maintained volumetric porosity above 97 percent and C6 viability at day 14 between about 100 and 106 percent. In contrast, the crater modified two dimensional mats by Hwang et al.^74^ reported only crater area porosity without volumetric porosity, compressive mechanics, 24 h PBS uptake, or 14-day viability, which limits direct like for like comparison of three-dimensional transport and mechanics. The three-dimensional wet electrospun PCL/gelatin plus SPION scaffolds by Bakhtiary et al.^81^ achieved similar volumetric porosity and a substantially higher compressive modulus of about 710 ± 200 kPa. Their PBS swelling was measured at 2 h and exceeded 2000 percent, a different timepoint and magnitude than our 24 h uptake, so we annotate this in Table 5 rather than forcing a direct numeric comparison. Finally, Du et al.^82^ evaluated two dimensional PCL/gelatin/arbutin membranes and reported that 24 h water absorption increased with arbutin content, but the exact percentages were not tabulated. Accordingly, Table 5 marks this metric as not reported while noting the trend. Altogether, by restricting the table to endpoints quantified in this work and annotating methodological and timepoint differences, we provide a transparent, quantitative side by side view showing that the present three-dimensional scaffold uniquely combines ultra high porosity, kilopascal range stiffness, tunable hydration, and sustained day 14 viability in a single platform, thereby addressing the structural and functional gaps of earlier PCL/gelatin systems.

**Table 5:** Quantitative side‑by‑side comparison with PCL/gelatin scaffolds reported in the literature.

Study	Scaffold type	Porosity (%)	Compressive modulus (kPa)	PBS uptake (%)	Cell viability day 14 (%)	Cell type	Notes	Refs.
This study (70:30–10 min)	3D PCL/gelatin; centrifugal wet‑electrospun	98.1 ± 1.9 (ethanol displacement)	53.0 ± 2.0	30.0 ± 1.0 (24 h, PBS)	100.4	C6 glial	Direct measurements; methods in Sect. “Porosity measurements”–“Mechanical evaluation”	–
This study (60:40–10 min)	3D PCL/gelatin; centrifugal wet‑electrospun	97.3 ± 1.1 (ethanol displacement)	24.7 ± 1.5	40.0 ± 1.0 (24 h, PBS)	105.9	C6 glial	Direct measurements; methods in Sect. “Porosity measurements”–“Mechanical evaluation”	–
Hwang et al	2D cratered PCL/gelatin mats	NR (SEM crater‑area porosity: 45.6–52.2%)	Not reported	Not reported	Not reported (7‑day MTS only)	hMSC	Area‑porosity within crater region reported; no volumetric porosity/mechanics	^74^
Bakhtiary et al	3D wet‑electrospun PCL/gelatin + SPION	98.0 ± 0.4 (ethanol displacement)	710 ± 200	> 2000 (2 h, PBS)	Not reported (1–7 d MTT)	OE‑MSCs	Compression E reported as 0.71 ± 0.2 MPa; PBS swelling after 2 h	^81^
Du et al	2D PCL/gelatin/arbutin membranes	Not reported	Not reported	Trend only (PCL/Gelatin/A > PCL/G) at 24 h	Not reported (24–72 h CCK‑8)	NIH‑3T3 fibroblasts	Water absorption method given; values plotted but not tabulated	^82^

### Conclusion and future perspective

### Future perspectives

While the current findings are promising, additional research is essential to fully validate the clinical potential and biological relevance of these 3D PCL/gelatin scaffolds. In particular, a deeper investigation into the cell–material interactions at the molecular level is warranted. Future studies should explore the activation of key signaling pathways, such as the integrin/FAK/ERK axis and PI3K/Akt/mTOR, which are known to regulate neural cell adhesion, proliferation, and axonal growth within ECM-like environments. Understanding how scaffold composition and architecture modulate these pathways would provide valuable mechanistic insights and enable rational scaffold design for enhanced neural regeneration.

Furthermore, preclinical in vivo studies in relevant nerve injury models are critical to assess the biocompatibility, degradation kinetics, immune response, and long-term functional efficacy of the optimized scaffolds. Such studies would establish the translational potential of these scaffolds and their suitability for clinical application in nerve repair and regeneration.

In addition, the versatility of the centrifugal wet electrospinning platform should be further leveraged to create multi-functional scaffolds. Incorporating bioactive cues—such as neurotrophic factors (e.g., NGF, BDNF), ECM peptides, or cytokines—could enhance scaffold bioactivity and promote more robust neural differentiation and axon guidance. Moreover, hybrid fabrication strategies, integrating wet electrospinning with additive manufacturing or micro-patterning, may enable spatially controlled fiber alignment and gradient architectures that better mimic the anisotropic nature of native neural tissues.

Finally, the adaptability of these scaffolds for other tissue engineering applications beyond neural repair should be explored. By fine-tuning polymer composition, fiber orientation, and mechanical properties, this platform holds promise for engineering scaffolds suitable for skin, muscle, and vascular regeneration, thereby broadening its biomedical utility.

### Conclusion

The findings of this study highlight the successful fabrication and optimization of 3D PCL/gelatin scaffolds for neural tissue engineering applications using centrifugal force-assisted wet electrospinning. Based on porosity analysis, both 70:30 and 60:40 PCL/gelatin scaffolds centrifuged at 10,000 rpm for 10 min exhibited excellent porosity values of 98.1 ± 1.9% and 97.3 ± 1.1%, respectively, which are highly favorable for facilitating cell infiltration and nutrient diffusion. In contrast, the 50:50 PCL/gelatin scaffold demonstrated the lowest porosity (< 90%) and a dense, fused morphology observed in SEM images, leading to its exclusion from further biological and mechanical evaluations.

Additionally, scaffolds fabricated at 5000 rpm exhibited severe structural deficiencies, including irregular macroscopic morphology, poor volume retention, and handling fragility, as confirmed in Fig. 2. These defects were attributed to insufficient centrifugal force during the fabrication process, resulting in inadequate fiber compaction and compromised scaffold integrity. Consequently, all 5000 rpm scaffolds were excluded from subsequent SEM, porosity, mechanical, and biological analyses to ensure that only mechanically robust and morphologically uniform scaffolds were evaluated.

The morphological characteristics assessed by SEM confirmed that the 60:40 scaffold exhibited the most homogeneous fiber distribution and ECM-like architecture, while the 70:30 scaffold provided a highly porous but slightly less uniform structure. Notably, PBS absorption tests revealed that the 60:40 scaffold centrifuged for 10 min exhibited the highest fluid uptake, attributed to its higher gelatin content and optimal pore architecture. In comparison, the 70:30 scaffold, despite its excellent porosity, showed lower PBS uptake due to increased fiber compaction and higher PCL content, which limits hydrophilic interactions. However, given that scaffold selection for neural tissue engineering must consider not only hydration but also mechanical stability and long-term bioactivity, PBS uptake alone was not considered the decisive factor.

Mechanical testing demonstrated that the 70:30 scaffold fabricated at 10,000 rpm for 10 min exhibited superior mechanical properties, with a tensile strength of 57.03 ± 1.50 kPa and a Young’s modulus of 53.00 ± 2.00 kPa, aligning well with the physiological range of neural tissues. This mechanical stability, combined with sufficient elasticity, provides an ideal environment to support dynamic cellular activities without compromising scaffold integrity.

Biological assays, including MTT, DAPI, H&E staining, and SEM imaging, further validated the biocompatibility and functionality of the scaffolds. The MTT assay confirmed sustained viability and proliferation of C6 glial cells, with an increase of + 4.30% on the 70:30–10 min scaffold and + 5.88% on the 60:40–10 min scaffold over 14 days. Although the 70:30–5 min scaffold exhibited the highest relative increase in cell viability (+ 12.16%), it showed inferior mechanical performance and structural uniformity compared to the 10 min variant. Consequently, considering the comprehensive balance of mechanical integrity, structural stability, and biological performance, the 70:30–10 min scaffold was selected for further morphological and histological analyses.

DAPI and H&E staining, along with SEM imaging, consistently demonstrated superior neural-like morphology, greater cell aggregation, and enhanced ECM deposition on the 70:30–10 min scaffold. These observations highlight that the optimal combination of mechanical integrity, porosity, and biochemical compatibility in the 70:30 scaffold more effectively supports neural-specific cellular behaviors.

In summary, this study demonstrates that scaffolds fabricated at 10,000 rpm provide superior performance across structural, mechanical, and biological parameters, whereas those fabricated at 5000 rpm are unsuitable due to poor reproducibility and stability. Among the 10,000 rpm scaffolds, the 70:30 PCL/gelatin scaffold centrifuged for 10 min emerged as the most promising candidate for neural tissue engineering, selected through a multi-parameter evaluation framework balancing mechanical strength, porosity, and bioactivity. These findings emphasize that scaffold optimization for neural tissue engineering requires a holistic approach, integrating structural, mechanical, and biological considerations to achieve clinically relevant outcomes.

## Data Availability

The datasets used and/or analysed during the current study available from the corresponding author on reasonable request.
